# Navigating Cross-Border E-Commerce: Prioritizing Logistics Partners with Hybrid MCGDM

**DOI:** 10.3390/e27080876

**Published:** 2025-08-19

**Authors:** Xingyu Ma, Chuanxu Wang

**Affiliations:** School of Economics and Management, Shanghai Maritime University, Shanghai 201306, China; 202130710069@stu.shmtu.edu.cn

**Keywords:** logistics suppliers selection, multiple criteria group decision making, heterogeneous data, cloud Bhattacharyya distance, cloud TOPSIS

## Abstract

As global e-commerce expands, efficient cross-border logistics services have become essential. To support the evaluation of logistics service providers (LSPs), we propose **HD-CBDTOPSIS** (Technique for Order Preference by Similarity to Ideal Solution with heterogeneous data and cloud Bhattacharyya distance), a hybrid multi-criteria group decision-making (MCGDM) model designed to handle complex, uncertain data. Our criteria system integrates traditional supplier evaluation with cross-border e-commerce characteristics, using heterogeneous data types—including exact numbers, intervals, digital datasets, multi-granularity linguistic terms, and linguistic expressions. These are unified using normal cloud models (NCMs), ensuring uncertainty is consistently represented. A novel algorithm, improved multi-step backward cloud transformation with sampling replacement (IMBCT-SR), is developed for converting dataset-type indicators into cloud models. We also introduce a new similarity measure, the Cloud Bhattacharyya Distance (CBD), which shows superior discrimination ability compared to traditional distances. Using the coefficient of variation (CV) based on CBD, we objectively determine criteria weights. A cloud-based TOPSIS approach is then applied to rank alternative LSPs, with all variables modeled using NCMs to ensure consistent uncertainty representation. An application case and comparative experiments demonstrate that HD-CBDTOPSIS is an effective, flexible, and robust tool for evaluating cross-border LSPs under uncertain and multi-dimensional conditions.

## 1. Introduction

Under the development opportunities presented by economic globalization, industrial digitization, and the Belt and Road Initiative, cross-border e-commerce integrates both e-commerce and international trade, which provides consumers with a wider variety of commodities and a more convenient way of shopping. Therefore, it has become an emerging format in international trade and a significant driving force of national economic growth. According to a report released by Statista in 2024 (Cross-border e-commerce in North America), the global cross-border e-commerce transaction volume approached 190.1 trillion dollars in 2023, and is expected to reach 290.2 trillion dollars by 2030. The favorable development trend and enormous market potential have attracted world-renowned e-commerce platforms represented by Alibaba, JingDong, Amazon, etc., to actively expand their cross-border e-commerce businesses. At the same time, an increasing number of overseas suppliers sell high-quality foreign products to end consumers through cross-border e-commerce platforms.

Compared with traditional trade forms, cross-border e-commerce greatly improves the cross-border operation efficiency of business flow, capital flow, and information flow via computer network equipment, while logistics must be provided by real logistics providers. Therefore, the vigorous development of cross-border e-commerce relies on efficient and reliable cross-border logistics services. At present, cross-border logistics involves multiple stages, including domestic warehousing and packaging, cross-border transportation, customs clearance, overseas delivery, and after-sales support. Complex intermediate links make cross-border logistics management more challenging. Any changes in links can bring potential risks to the entire supply chain network, even leading to problems, such as high return rates and rising costs. The trend of modern enterprise management is to reduce the number of suppliers and establish a stable cooperative relationship based on mutual trust and mutual benefit, which exacerbates the risk in the selection of cross-border LSPs. Moreover, when consumers purchase cross-border products, the most important factors are product cost performance and delivery speed. Due to the differences in geographical and cultural distances, overseas suppliers may make incorrect decisions on product pricing or logistics resource allocation in situations of demand information asymmetry, which results in reducing consumer satisfaction and overall supply chain performance. How to select the appropriate logistics service providers (LSPs) will play a crucial role in the development of cross-border e-commerce enterprises.

Supplier selection or third-party LSP selection is a typical multi-criteria decision making (MCDM) problem. Hausman et al. [1] took a supply chain perspective to consider logistics performance, selecting import and export activities’ cost, time, and complexity as criteria. Zeng et al. [2] focused on the LSP evaluation from financial and non-financial perspectives. Based on supply chain risk, Chen and Wu [3] proposed 17 criteria from six dimensions: cost, quality, deliverability, technology, productivity and service. Yao [4] systematically evaluated cross-border e-commerce supply chain partners from the perspectives of environmental risk, commodity risk, venture risk, logistics risk. Kumar et al. [5] and Hendiani and Walther [6] established sustainable supplier evaluation criteria systems, including economic, social, and environmental factors, such as quality, energy use, pollution control, and reputation. Alkhatib et al. [7] highlighted that internet-based technology and cooperation relationship are key factors reflecting the LSP’s resource and capability.

After constructing the evaluation criteria system that has been fully considered in many aspects, many scholars have designed the criteria weight and alternatives ranking methods. For selecting third-party LSP of agricultural products, Huang et al. [8] established an evaluation criteria system with 13 indicators including customer satisfaction, in which quantitative indicators were modeled with fuzzy error functions according to benefit type and cost type, respectively. Comprehensive loss, overall coordination, and final ranking were calculated based on error loss. Li et al. [9] screened 11 listed logistics enterprises and obtained eight quantitative criteria from financial statements. After max-min normalization of the evaluation data, the criteria weight was calculated by entropy method. Grey relational analysis and TOPSIS were used to rank the innovation performance of enterprises, respectively. In terms of score variance, TOPSIS had greater discrimination ability. Their studies [8,9] did not consider uncertain information and group decision-making. Supplier selection as a complex decision-making process, is easily influenced by the subjectivity and vagueness of human judgment. Li [10] integrated rough set theory and gray theory with TOPSIS to evaluate LSP. Hendiani and Walther [6] mapped the linguistic terms obtained through expert consultation to interval intuitionistic fuzzy sets as criteria weights. Lin and Tseng [11] applied interval-valued triangular fuzzy numbers to represent the evaluation values and criteria weights for qualitative criteria. Qin et al. [12] used 2-order additive fuzzy measures to describe independence, redundancy, and complementarity correlation among criteria, and aggregated the evaluation of correlative criteria based on Choquet integral. Wang et al. [13] and Ramakrishnan and Chakraborty [14] converted linguistic variables into cloud models to completely reflect the randomness and fuzziness of qualitative concepts. Ghadikolaei et al. [15] combined extended hesitant fuzzy linguistic information and VIKOR method for group decision-making. Under group consensus, Kar [16] adopted fuzzy AHP for criteria weight determination and fuzzy NN for supplier prioritization. Some researchers only employ quantitative criteria [9,10,12,17], while others only use qualitative criteria [6,11,13,14,15,16]. However, decision makers with different experiences and preferences may provide diverse types of evaluation information, such as exact numbers and natural languages. Furthermore, different criteria are appropriate for using heterogeneous data representations. Li et al. [18] used the TODIM method to process heterogeneous evaluation information including crisp numbers, interval numbers, and linguistic terms. Yang et al. [19] comprehensively considered heterogeneous data evaluation, representing crisp values, interval numbers, statistical data, and linguistic terms as normal cloud models (NCMs).

The small-batch, high-frequency cross-border e-commerce business model places higher requirements on the reliability and flexibility of the supply chain. Nevertheless, in the field of cross-border LSP prioritization, the application of MCDM methods is still very limited. (1) Most existing studies only consider either quantitative criteria or qualitative criteria and fail to fully extract decision-making information from various data types. The single data format may lead to information loss and result inaccuracy, ultimately affecting the quality of decision-making. (2) The methods of handling quantitative criteria are inadequate. Although exact numbers and interval numbers have been extensively studied, there is little discussion on datasets. Yang et al. [19] represented statistical data as NCMs, in which the mean, variance and zero are simply taken as numerical characteristics. There is still room for further research to explore the randomness and vagueness of the data of dataset type. (3) The evaluation result obtained by traditional TOPSIS, VIKOR or other methods is a definite ranking. Considering various uncertainty of input data, the evaluation result should reflect uncertainty. (4) The methods for determining criteria weights are primarily divided into subjective weighting and objective weighting. Subjective weighting methods, such as Delphi and AHP, rely on the experts’ judgment and lack objective data support, which not only increases the burden on decision-makers but also introduces subjectivity and uncertainty into the evaluation results. Objective weighting methods with strong discrimination abilities are worth developing.

On the one hand, building upon the literature review of 217 papers, this work integrates traditional supplier selection criteria and the cross-border e-commerce transaction process to establish a comprehensive evaluation criteria system for cross-border LSPs from a risk perspective. The criteria selected from four aspects: logistics quality, logistics cost, logistics capability, and development potential. On the other hand, a novel multi-criteria group decision making method (TOPSIS with heterogeneous data and cloud Bhattacharyya distance, abbreviated as *HD-CBDTOPSIS*), is proposed. In summary, the major contributions are summarized as follows.

(a) **Flexible Integration of Diverse Data Types**: We consider both quantitative and qualitative criteria within a group decision-making framework by accommodating heterogeneous data formats—including exact numbers, intervals, digital datasets, multi-granularity linguistic terms, and general linguistic expressions. This enables experts to express their opinions with greater flexibility and realism.
(b) **Unified Representation through Normal Cloud Models (NCMs)**: We develop a comprehensive mechanism to convert all types of evaluation data into NCMs. Notably, we propose a novel Improved Multi-step Backward Cloud Transformation with Sampling Replacement (IMBCT-SR) algorithm specifically for dataset-type indicators. Its performance advantages are validated through comparative experiments (Figure 2).
(c) **Enhanced Cloud-Based TOPSIS for Decision Prioritization**: We apply a cloud-enhanced TOPSIS method to rank cross-border LSPs. Unlike conventional approaches, our method models all key elements—such as weights, ideal solutions, and rankings—using NCMs, allowing uncertainty to be fully retained throughout the evaluation process.
(d) **Objective Weighting and Advanced Similarity Measurement**: Criteria weights are determined using the coefficient of variation (CV), ensuring an objective influence assessment. In addition, we propose a new similarity measure called the Cloud Bhattacharyya Distance (CBD) to compare NCMs. CBD is shown to satisfy standard distance properties and demonstrates superior discrimination ability over Wasserstein Distance (WD) [20] (Table 3 and Figure 5).

The rest of this paper is organized as follows: Section 2 briefly introduces the methods, including cloud model theory, cloud generator, linguistic information, and group decision-making based on heterogeneous data. Section 3 derives Bhattacharyya distance of NCMs and compares CBD with WD. Section 4 presents the framework of HD-CBDTOPSIS. An application example and comparative analysis are shown in Section 5. Finally, we summarize contributions, limitations and future research directions.

## 2. Preliminaries

This section begins by outlining the fundamental concepts and operations of NCMs. Following this, the essential concepts and representation methods for linguistic information are presented. Finally, we describe the methods for converting heterogeneous data into NCM representations. These foundational elements establish the theoretical basis for the representation (Section 2.2 and Section 2.3), transformation (Section 2.4), and computation (Section 2.1) of heterogeneous data using NCMs.

### 2.1. Cloud Model Theory

Much real-world decision-making information is too complex and vague to be expressed as precise values. Instead, it is often described using linguistic terms, such as {‘very low’, ‘low’, ‘medium’, ‘high’, ‘very high’}. Aiming to transform uncertainty of qualitative concepts into quantitative analysis, the cloud model was first proposed in [21] based on probability theory and fuzzy set theory. Normal cloud models (NCMs) use random variables following normal distributions and Gaussian membership functions to articulate both randomness and fuzziness. Subsequently, we elaborate the basic definitions and operation rules of NCMs.

**Definition** **1** ([21])**.** *Let $U=[u_{min},u_{max}]$ be the universe of discourse and T be a qualitative concept in U. The numerical characteristics of T are described from three aspects: Expectation (Ex), Entropy (En), and hyper-entropy (He), where En≥0 and He≥0. If x∈U is a random instance of T which satisfies $x∼N(Ex,En^{\prime 2})$ and $En^{\prime }∼N\left(En,He^{2}\right)$, $\mu_{T}\left(x\right)\in \left[0,1\right]$, the certainty degree of x∈T, is defined as follows:*

(1) $$\mu_{T}\left(x\right)=\exp\left(-\frac{{\left(x-Ex\right)}^{2}}{2{\left(En^{\prime }\right)}^{2}}\right),$$

The distribution of *x* is referred to as an NCM, denoted as $Y_{T}=\left(Ex,En,He\right)$, and $\left(x,\mu_{T}\left(x\right)\right)$ represents a cloud droplet. The mathematical expectation Ex reflects the central location of the NCM. The entropy En is similar to standard deviation, measuring both randomness and fuzziness inherent in linguistic terms. Fuzziness relates to the range of values for *x*, such as $\left[Ex-3En^{\prime },Ex+3En^{\prime }\right]$. Randomness relates to different perceptions of decision-makers. For example, one expert considers that the rating of ‘high’ is around 7 and the membership of 6.5 belonging to ‘high’ is 0.8, while another thinks that the rating of ‘high’ is around 8 and the membership of 6.5 belonging to ‘high’ is 0.6. The NCM makes the membership follow a probability distribution, which alleviates the information aggregation distortion caused by non-uniform cognition to some extent. The entropy of entropy He corresponds to the uncertainty of En, indirectly reflecting the thickness of an NCM. The larger the He, the thicker the NCM.

**Definition** **2** ([22,23,24])**.** *Given two arbitrary NCMs $Y_{T_{1}},Y_{T_{2}}\in U$, $Y_{T_{1}}=\left(Ex_{1},En_{1},He_{1}\right)$ and $Y_{T_{2}}=\left(Ex_{2},En_{2},He_{2}\right)$, the basic operation rules are defined as follows:*

(2) $$\begin{array}{cc} \mathrm{addition}:Y_{T_{1}}+Y_{T_{2}} & =\left(Ex_{1}+Ex_{2},\sqrt{{\left(En_{1}\right)}^{2}+{\left(En_{2}\right)}^{2}},\sqrt{{\left(He_{1}\right)}^{2}+{\left(He_{2}\right)}^{2}}\right), \end{array}$$

(3) $$\begin{array}{cc} \mathrm{subtraction}:Y_{T_{1}}-Y_{T_{2}} & =\left(Ex_{1}-Ex_{2},\sqrt{{\left(En_{1}\right)}^{2}+{\left(En_{2}\right)}^{2}},\sqrt{{\left(He_{1}\right)}^{2}+{\left(He_{2}\right)}^{2}}\right), \end{array}$$

(4) $$\begin{array}{cc} \mathrm{multiplication}:Y_{T_{1}}\times Y_{T_{2}} & =\left(Ex_{1}Ex_{2},\sqrt{{\left(En_{1}Ex_{2}\right)}^{2}+{\left(En_{2}Ex_{1}\right)}^{2}},\sqrt{{\left(He_{1}Ex_{2}\right)}^{2}+{\left(He_{2}Ex_{1}\right)}^{2}}\right), \end{array}$$

(5) $$\begin{array}{cc} \mathrm{division}:\;\;Y_{T_{1}}/Y_{T_{2}} & =\left(\frac{Ex_{1}}{Ex_{2}},\sqrt{{\left(\frac{En_{1}}{Ex_{2}}\right)}^{2}+{\left(\frac{En_{2}Ex_{1}}{{\left(Ex_{2}\right)}^{2}}\right)}^{2}},\sqrt{{\left(\frac{He_{1}}{Ex_{2}}\right)}^{2}+{\left(\frac{He_{2}Ex_{1}}{{\left(Ex_{2}\right)}^{2}}\right)}^{2}}\right). \end{array}$$

**Remark** **1.** *$Y_{T_{1}}\pm 0=Y_{T_{1}}$;   $Y_{T_{1}}+Y_{T_{2}}=Y_{T_{2}}+Y_{T_{1}}$; $\left(Y_{T_{1}}+Y_{T_{2}}\right)+Y_{T_{3}}=Y_{T_{1}}+\left(Y_{T_{2}}+Y_{T_{3}}\right)$; $\mu Y_{T_{1}}=(\mu Ex_{1},|\mu |En_{1},|\mu |He_{1}),$ $Y_{T_{1}}^{\lambda }=\left(Ex_{1}^{\lambda },\sqrt{\lambda }Ex_{1}^{\lambda -1}En_{1},\sqrt{\lambda }Ex_{1}^{\lambda -1}He_{1}\right)$,  μ,λ∈R.*

**Definition** **3** ([24,25])**.** *Given two arbitrary NCMs $Y_{T_{1}},Y_{T_{2}}\in U$, $Y_{T_{1}}=\left(Ex_{1},En_{1},He_{1}\right)$ and $Y_{T_{2}}=\left(Ex_{2},En_{2},He_{2}\right)$, the comparison rules are given as follows:*

*(a)*  *If $Ex_{1}>Ex_{2}$, then $Y_{T_{1}}>Y_{T_{2}}$;*
*(b)*  *If $Ex_{1}=Ex_{2}$ and $En_{1}<En_{2}$, then $Y_{T_{1}}>Y_{T_{2}}$;*
*(c)*  *If $Ex_{1}=Ex_{2}$, $En_{1}=En_{2}$, and $He_{1}<He_{2}$, then $Y_{T_{1}}>Y_{T_{2}}$;*
*(d)*  *If and only if $Ex_{1}=Ex_{2}$, $En_{1}=En_{2}$, and $He_{1}=He_{2}$, then $Y_{T_{1}}=Y_{T_{2}}$.*

**Definition** **4** ([22,23,24])**.** *Given N arbitrary NCMs $Y_{T_{i}}=\left(Ex_{i},En_{i},He_{i}\right)\in U\;\left(i=1,2,\ldots ,N\right)$, the cloud synthetic operator $f_{CS}:\;Y_{T}^{N}\to Y_{T}$ is given as follows:*

(6) $$f_{CS}\left(Y_{T_{1}},Y_{T_{2}},\ldots ,Y_{T_{N}}\right)=\left(\frac{1}{N}\sum_{i=1}^{N}Ex_{i},\frac{1}{6}\left(\max_{i}\left(Ex_{i}+3En_{i}\right)-\min_{j}\left(Ex_{j}-3En_{j}\right)\right),\sqrt{\sum_{i=1}^{N}{\left(He_{i}\right)}^{2}}\right).$$

**Definition** **5** ([22,23,24])**.** *Given N arbitrary NCMs $Y_{T_{i}}=\left(Ex_{i},En_{i},He_{i}\right)\in U\;\left(i=1,2,\ldots ,N\right)$, each of which is assigned a weight $w_{i}$, the cloud weighted average operator $f_{CW\;A}:\;Y_{T}^{N}\to Y_{T}$ is given as follows:*

(7) $$f_{CW\;A}\left(Y_{T_{1}},Y_{T_{2}},\ldots ,Y_{T_{N}}\right)=\sum_{i=1}^{N}w_{i}Y_{T_{i}}/\sum_{i=1}^{N}w_{i}.$$

If $w_{i}\in \left[0,1\right]$ is a real number for all i=1,2,…,N, and $\sum_{i=1}^{N}w_{i}=1$, Equation (7) is simplified as

(8) $$f_{CW\;A}\left(Y_{T_{1}},Y_{T_{2}},\ldots ,Y_{T_{N}}\right)=\sum_{i=1}^{N}w_{i}Y_{T_{i}}=\left(\sum_{i=1}^{N}w_{i}Ex_{i},\sqrt{\sum_{i=1}^{N}{\left(w_{i}En_{i}\right)}^{2}},\sqrt{\sum_{i=1}^{N}{\left(w_{i}He_{i}\right)}^{2}}\right).$$

The synthetic NCM treats all NCMs equally important, while the weighted average NCM can balance NCMs with different contributions. Furthermore, both En and He of the synthetic NCM exceed those of each single NCM, indicating that it can cover a wider information scope.

### 2.2. Cloud Generator

**Figure 1:**
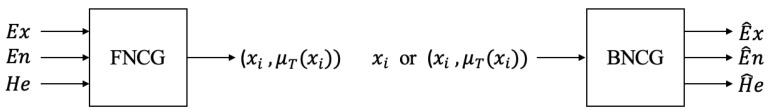
Forward normal cloud generator (FNCG) and backward normal cloud generator (BNCG).

The objective and interchangeable conversion between linguistic terms and quantitative values is accomplished through forward and backward cloud generator, as shown in Figure 1. Forward cloud generator produces cloud droplets (quantitative) based on the numerical characteristics that represent linguistic concepts (qualitative), with its algorithm provided in Algorithm 1.

**Algorithm 1:** The algorithm of FNCG.
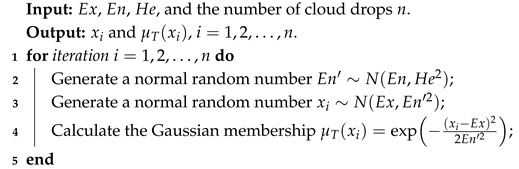


Backward cloud generator uses the statistics of sampled cloud droplets to estimate the numerical characteristics, which mainly categorized into those requiring membership information [26,27,28] and those not relying on that [29,30]. A multi-step backward cloud transformation algorithm based on sampling with replacement (MBCT-SR) divides *n* cloud droplets into *m* groups, each containing *r* cloud droplets. Experiments demonstrate that MBCT-SR outperforms other BNCG algorithms [31,32]. Xu et al. [32] concluded that *n* and *m* just affect the convergence of estimators, while the absolute error reaches a minimum when *r* exhibits a power-law relationship with He/En. In order to improve the accuracy of parameter estimation, we propose an improved MBCT-SR (IMBCT-SR) as listed in Algorithm 2, where *m* is determined by Sturges’ formula [33], *r* is initially estimated using [n/m] and then updated based on the power-law relationship.

**Figure 2:**
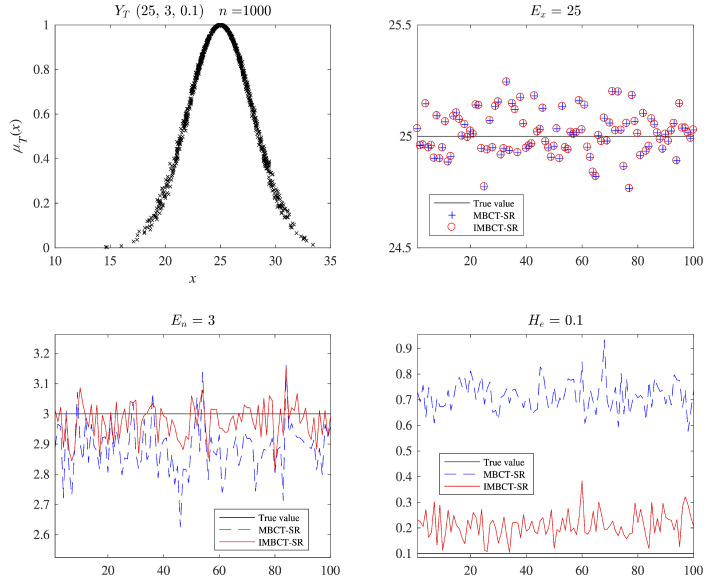
An example of the accuracy comparison between IMBCT-SR and MBCT-SR.

**Figure 3:**
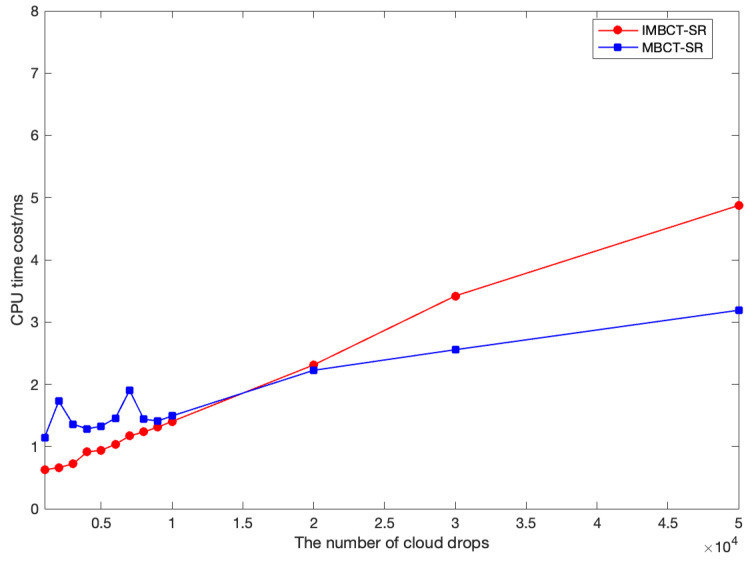
An example of the CPU consumption time comparison between IMBCT-SR and MBCT-SR.

Experimental results show that IMBCT-SR can estimate the values of En and He more accurately than MBCT-SR. For example, using the FNCG to randomly generate 1000 cloud droplets with parameters (25,3,0.1), we estimate the cloud model parameters from these cloud droplets by IMBCT-SR and MBCT-SR, respectively. A detailed accuracy comparison for 100 runs is shown in Figure 2. Additionally, to illustrate the effectiveness of IMBCT-SR, we further compare the runtime of IMBCT-SR and MBCT-SR under varying data scales. The number of cloud droplets *n* is ranged from 1000 to 50,000. For each *n*, both algorithms are executed 50 times, and average CPU consumption is recorded, as shown in Figure 3. When the number of cloud droplets n< 10,000, the computing time is less than 2ms, which fully meets the computational time requirements for almost all real-world scenarios. Both IMBCT-SR and MBCT-SR exhibit a positive correlation between runtime and the number of cloud droplets, which generally aligns with their theoretical linear time complexity O(n). MBCT-SR demonstrates noticeable computational redundancy at smaller data scales (n< 20,000) due to its fixed grouping strategy, resulting in longer computing time compared to IMBCT-SR. In contrast, IMBCT-SR dynamically adjusts the grouping parameter (*m*) and sampling size (*r*), increasing sampling magnitude only when processing low-noise data (small He/En). This adaptive mechanism effectively avoids the computational overhead of repeated grouping, improving parameter estimation accuracy and computing efficiency. It is worth noting that when dealing with extremely large-scale data (n> 50,000), IMBCT-SR’s iterative sampling process incurs additional computational burden. However, compared to conventional algorithms with time complexity $O(n^{2})$ or O(nlogn), both IMBCT-SR and MBCT-SR demonstrate excellent practicability.

**Algorithm 2:** The algorithm of IMBCT-SR.
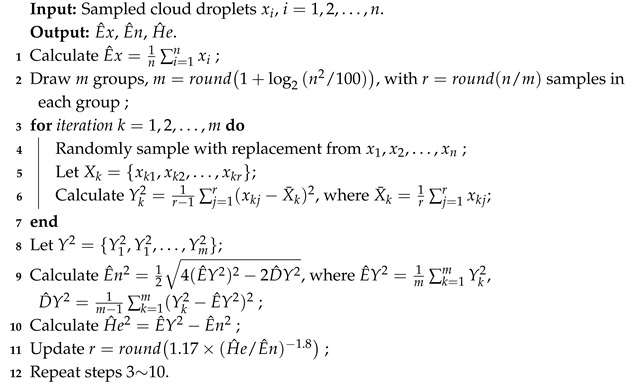


### 2.3. Linguistic Information

#### 2.3.1. Linguistic Term

NCMs are commonly adopted for modeling linguistic terms. There are alternative methods for constructing standard clouds including the golden section method [27,34] and the theta scaling method [35]. The golden section method limits the set of linguistic terms with only five members [13]. In order to achieve exponential growth of semantic distance and better fit the actual decision-making scenarios, the theta scaling method extends to −k∼k scale integrating the advantages of exponential scale [36]. In this work, we adopt the theta scaling method which is elaborated in Definition 6 and Algorithm 3.
**Algorithm 3:** The algorithm of theta scaling method.
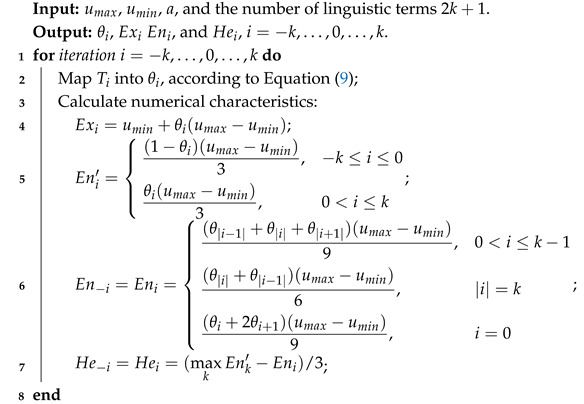

**Definition** **6** ([13])**.** Given a linguistic term set $T=\{T_{i}|i=-k,\ldots ,0,\ldots ,k,k\in N^{*}\}$, $T_{i}$ is mapped into $\theta_{i}$ by the linguistic scale function f as follows:

(9) $$\theta_{i}=f\left(T_{i}\right)=\left\{\begin{array}{cc} \frac{a^{k}-a^{-i}}{2a^{k}-2}, & -k\leq i\leq 0\\ \frac{a^{k}+a^{i}-2}{2a^{k}-2}, & 0<i\leq k \end{array}\right.$$

*where a is a hyper-parameter, likely to fall within the interval [1.36,1.4], according to previous experimental studies. Moreover, a can also subjectively be assigned the value of 1.37 [35,36]. Obviously, $\left\{\theta_{i}\right\}$ is a monotonically increasing sequence. if −k≤i≤0, then $0\leq \theta_{i}\leq 0.5$; if 0<i≤k, then $0.5<\theta_{i}\leq 1$; if i+j=0, then $\theta_{i}+\theta_{j}=1$.*

**Example** **1.** *Let the linguistic term set $T=\{T_{-3}=\mathrm{none},T_{-2}=\mathrm{very}\;\mathrm{low},T_{-1}=\mathrm{low}$, $T_{0}=\mathrm{medium},T_{1}=\mathrm{high},T_{2}=\mathrm{very}\;\mathrm{high},T_{3}=\mathrm{perfect}\}$ and the universe U=[0,10], the results of encoding T into NCMs by theta scaling method with a=1.37 are shown in Table 1.*

**Figure 4:**
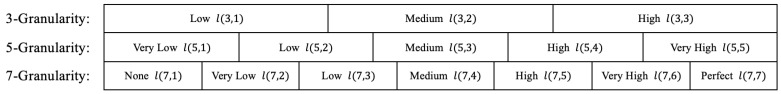
Linguistic term sets with different granularities.

Taking into account experience and preference, different decision makers within the same qualitative assessment or even one decision maker for different qualitative assessments tend to divide the semantic space into different granularities. Consequently, the linguistic terms used do not adhere to a unified linguistic term set, as shown in Figure 4. Allowing decision makers to flexibly choose linguistic terms with granularities that match their preferences can better reflect the nuances of real-world decisions, improving the authenticity and effectiveness of evaluation results. The utilization of different parameters *k* in the theta scaling method can generate different multi-granularity linguistic term sets.

#### 2.3.2. Linguistic Expression

Although an ambiguous linguistic term can be modeled as an NCM $Y_{T}\left(Ex,En,He\right)$, experts may have difficulty in providing a single linguistic term to express their qualitative opinions in complex decision situations. Instead, they may hesitate among several linguistic terms or seek composite linguistic expressions, such as ‘between low and medium’ or ‘at least high’, which are not predefined in the linguistic term set. To address such issue, Huang and Yang [37] introduced a concept of hesitant cloud linguistic term set (HCLTS) based on HFLTS [38].

**Definition** **7** ([23,24,38])**.** Given a linguistic term set $T=\{T_{i}|i=-k,\ldots ,0,\ldots ,k,k\in N^{*}\}$, a context-free grammar $G_{T}=\left(V_{N},V_{T},I,P\right)$ is defined as follows:

$$\begin{array}{cc} V_{N}=\{ & <\mathrm{primary}\;\mathrm{term}>,\;<\mathrm{composite}\;\mathrm{term}>,\;<\mathrm{unary}\;\mathrm{relation}>,\\ \; & <\mathrm{binary}\;\mathrm{relation}>,\;<\mathrm{conjunction}>\};\\ V_{T}=\{ & \mathrm{lower}\;\mathrm{than},\;\mathrm{greater}\;\mathrm{than},\;\mathrm{at}\;\mathrm{most},\;\mathrm{at}\;\mathrm{least},\;\mathrm{between},\;\mathrm{and},T_{-k},\ldots ,T_{0},\ldots ,T_{k}\};\\ P=\{ & I::=\left\{<\mathrm{primary}\;\mathrm{term}>|<\mathrm{composite}\;\mathrm{term}>\right\}\in V_{N},\\ \; & <\mathrm{primary}\;\mathrm{term}>::=\;T_{-k}\left|\ldots \right|T_{0}\left|\ldots \right|T_{k},\\ & <\mathrm{composite}\;\mathrm{term}>::=\;<\mathrm{unary}\;\mathrm{relation}><\mathrm{primary}\;\mathrm{term}>\;|\\ \; & \;\;\;<\mathrm{binary}\;\mathrm{relation}><\mathrm{primary}\;\mathrm{term}><\mathrm{conjunction}><\mathrm{primary}\;\mathrm{term}>,\\ \; & <\mathrm{unary}\;\mathrm{relation}>::=\;\mathrm{lower}\;\mathrm{than}\;|\;\mathrm{greater}\;\mathrm{than}\;|\;\mathrm{at}\;\mathrm{most}\;|\;\mathrm{at}\;\mathrm{least},\\ \; & <\mathrm{binary}\;\mathrm{relation}>::=\;\mathrm{between},\\ & <\mathrm{conjunction}>::=\;\mathrm{and}\}. \end{array}$$

**Definition** **8** ([23,24,37])**.** Given a orderly finite linguistic term set $T=\{T_{i}|i=-k,\ldots ,k,k\in N^{*}\}$, where $T_{i}$ is encoded into an NCM $Y_{T_{i}}=\left(Ex_{i},En_{i},He_{i}\right)$, an HCLTS $H_{T}$ is defined as a orderly finite and consecutive subset of T.

The expressions generated by context-free grammar $G_{T}$ are more aligned with human subjective perceptions, but they require conversion into HCLTSs for computation. Note that the expression domain generated by $G_{T}$ is $T_{ll}$. A transformation function $f_{G_{T}}:\;T_{ll}\to H_{T}$ is proposed in Definition 9.

**Definition** **9** ([23,24,38])**.** *Given a linguistic term set T along with its context-free grammar $G_{T}$ and HCLTS $H_{T}$, the function $f_{G_{T}}$ that transforms $G_{T}$ into $H_{T}$ follows different production rules according to the semantics of the linguistic expressions.*

*(a)*  *$f_{G_{T}}\left(T_{i}\right)=\left\{T_{i}|T_{i}\in T\right\}$;*
*(b)*  *$f_{G_{T}}\left(\mathrm{lower}\;\mathrm{than}\;T_{i}\right)=\left\{T_{j}|T_{j}<T_{i}\;\mathrm{and}\;T_{j}\in T\right\}$;*
*(c)*  *$f_{G_{T}}\left(\mathrm{greater}\;\mathrm{than}\;T_{i}\right)=\left\{T_{j}|T_{j}>T_{i}\;\mathrm{and}\;T_{j}\in T\right\}$;*
*(d)*  *$f_{G_{T}}\left(\mathrm{at}\;\mathrm{most}\;T_{i}\right)=\left\{T_{j}|T_{j}\leq T_{i}\;\mathrm{and}\;T_{j}\in T\right\}$;*
*(e)*  *$f_{G_{T}}\left(\mathrm{at}\;\mathrm{least}\;T_{i}\right)=\left\{T_{j}|T_{j}\geq T_{i}\;\mathrm{and}\;T_{j}\in T\right\}$;*
*(f)*  *$f_{G_{T}}\left(\mathrm{between}\;T_{i}\;\mathrm{and}\;T_{j}\right)=\left\{T_{k}|T_{i}\leq T_{k}\leq T_{j}\;\mathrm{and}\;T_{k}\in T\right\}$.*

**Example** **2.** *Two different HCLTSs in Example 1 might be $H_{T}^{1}=\left\{T_{-1},T_{0}\right\}$, and $H_{T}^{2}=\left\{T_{1},T_{2},T_{3}\right\}$, corresponding to linguistic expressions ‘between low and medium’ and ‘at least high’, respectively.*

### 2.4. Group Decision Making Based on Heterogeneous Data

Experts based on different knowledge background and cognitive preferences may provide diverse types of evaluation information, such as exact numbers, interval numbers, multi-granularity linguistic terms, or linguistic expressions. Therefore, in order to process heterogeneous data in group decision making for qualitative indicator evaluation, the conversion methods are summarized in Table 2.

Upon completion of unifying expert evaluations into NCMs, determining experts’ weights becomes an indispensable part in the group decision-making process. Different weight vectors can lead to different results. However, in previous research, consideration was rarely given to experts’ weights being either assumed to be same or predetermined [39,40]. To avoid subjectivity, it is necessary to consider the quality of decision information. In other words, different weights should be assigned to different decision information. Yang et al. [23] proposed a dynamic weights assignment algorithm, combining both uncertainty degree and consistency degree of expert evaluations. The uncertainty degree (UD) is measured by En and He of NCMs, while consistency degree (CD) refers to the consensus bias between individual evaluations and collective evaluations. We defined UD and CD, respectively, as Equations (10) and (11).

(10) $$\begin{array}{cc} UD_{i} & =\min\left(1,\frac{3(En_{i}+3He_{i})}{10}\right) \end{array}$$

(11) $$\begin{array}{cc} CD_{i} & =\min\left(1,3\times \left|Ex_{i}-\frac{1}{p}\sum_{j=1}^{p}Ex_{j}\right|/10\right) \end{array}$$

where $\left(Ex_{i},En_{i},He_{i}\right)$ represents *i*-th expert’s evaluation result.

Assuming there are *p* experts, the relative weight of each expert is determined by integrating UD and CD.

(12) $$w_{i}=\frac{\alpha \left(1-UD_{i}\right)+\left(1-\alpha \right)\left(1-CD_{i}\right)}{\sum_{i=1}^{p}\alpha \left(1-UD_{i}\right)+\left(1-\alpha \right)\left(1-CD_{i}\right)}$$

where α is a hyper-parameter controlling the importance of UD and CD to weights.

In conclusion, the group decision making process of qualitative indicators with heterogeneous data is described in Algorithm 4.
**Algorithm 4:** Group decision making process.
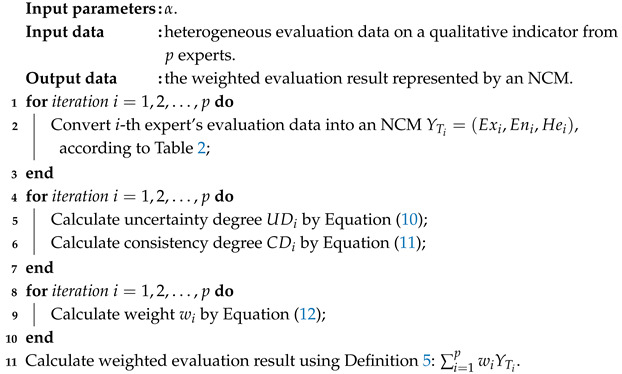


## 3. Dissimilarity Measures of NCMs Based on Bhattacharyya Distance

### 3.1. Introduction to Bhattacharyya Distance

Common dissimilarity measures between two discrete or continuous probability distributions are KL divergence, Wasserstein distance, Bhattacharyya distance, Hellinger distance, etc. Due to its inherent asymmetry and support sensitivity, KL divergence proves unsuitable for our cross-border e-commerce LSP evaluation framework, where multiple criteria contain either: (1) sparse expert ratings, or (2) highly divergent expert evaluations. Both scenarios will lead to metric failure. Wasserstein distance, also known as Earth-Mover distance, is defined as the minimum loss of moving one probability distribution to another. Xu and Yang [20] constructed an NCM similarity measure based on Wasserstein distance, using three numerical characteristics of the NCM. Mathematically, it essentially constitutes a Euclidean distance metric that captures geometric dissimilarity between numerical features. However, this approach exhibits significant limitations. For instance, Wasserstein distance between (3,1,0.1) and (4,1,0.1) is same as that between (9,1,0.1) and (10,1,0.1). Such mathematical equidistance fails to accurately reflect decision-makers’ cognitive patterns. In reality, experts’ psychological perception of evaluation scales like “very poor”, “poor”, “medium”, “good”, and “very good”, demonstrates distinct nonlinear characteristics, with semantic distances following gradient transition patterns. Bhattacharyya distance focuses on the distribution pattern and determines the similarity between two probability distributions by assessing the degree of overlap. In view of the fact that all evaluation data are within the same universes of discourse, Bhattacharyya distance, which is highly sensitive to overlapping information, is more conducive to improving discrimination than Wasserstein distance.

Taking continuous probability distributions as an example, Bhattacharyya distance is defined as follows [41]:

(13) $$BD\left(p\left(x\right),q\left(x\right)\right)=-\ln\int_{x}\;\sqrt{p(x)q(x)}dx,\;x\in R^{n};$$

For two multidimensional normal distributions p(x) and q(x), Bhattacharyya distance is derived as

(14) $$BD\left(p\left(x\right),q\left(x\right)\right)=\frac{1}{2}\ln\frac{\left|\sum \right|}{\sqrt{|\sum_{p}\sum_{q}|}}+\frac{1}{8}{\left(\mu_{p}-\mu_{q}\right)}^{T}\sum^{-1}\left(\mu_{p}-\mu_{q}\right);$$

where $\sum =\frac{1}{2}\left(\sum_{p}+\sum_{q}\right)$. $\mu_{p},\mu_{q}\in R^{n}$ and $\sum_{p},\sum_{q}\in R^{n\times n}$ are mean vectors and covariance matrices of p(x) and q(x).

For two one-dimensional normal distributions p(x) and q(x), Bhattacharyya distance is derived as

(15) $$BD\left(p\left(x\right),q\left(x\right)\right)=\frac{1}{2}\ln\frac{\sigma_{p}^{2}+\sigma_{q}^{2}}{2\sigma_{p}\sigma_{q}}+\frac{1}{4}\frac{{\left(\mu_{p}-\mu_{q}\right)}^{2}}{\sigma_{p}^{2}+\sigma_{q}^{2}},\;x\in R;$$

where $\mu_{p},\mu_{q}\in R$ represent means of p(x) and q(x), respectively. $\sigma_{p}^{2},\sigma_{q}^{2}\in R$ are variances of p(x) and q(x), respectively.

### 3.2. Bhattacharyya Distance of Two NCMs

Based on the conclusion that the expectation of normal cloud droplets is Ex, variance is $En^{2}+He^{2}$ given in the literature [42], we introduce Bhattacharyya distance, for the first time, into the NCM, according to Equation (15). We propose the following *extended definition*:

**Definition** **10.** *Given two arbitrary NCMs $Y_{T_{1}},Y_{T_{2}}\in U$, $Y_{T_{1}}=\left(Ex_{1},En_{1},He_{1}\right)$ and $Y_{T_{2}}=\left(Ex_{2},En_{2},He_{2}\right)$, their Bhattacharyya distance is*

(16) $$CBD\left(Y_{T_{1}},Y_{T_{2}}\right)=\frac{1}{2}\ln\frac{En_{1}^{2}+He_{1}^{2}+En_{2}^{2}+He_{2}^{2}}{2\sqrt{\left(En_{1}^{2}+He_{1}^{2}\right)\left(En_{2}^{2}+He_{2}^{2}\right)}}+\frac{1}{4}\frac{{\left(Ex_{1}-Ex_{2}\right)}^{2}}{En_{1}^{2}+He_{1}^{2}+En_{2}^{2}+He_{2}^{2}}.$$

It can be seen from Equation (16) that $CBD(Y_{T_{1}},Y_{T_{2}})$ satisfies the following properties:

(a) Non-negativity: $CBD(Y_{T_{1}},Y_{T_{2}})\geq 0$;
(b) Normalization: if $Y_{T_{1}}=Y_{T_{2}}$, then $CBD(Y_{T_{1}},Y_{T_{2}})=0$;
(c) Symmetry: $CBD\left(Y_{T_{1}},Y_{T_{2}}\right)=CBD\left(Y_{T_{2}},Y_{T_{1}}\right)$

**Proof.** 

(a) Based on mean inequality, $En_{1}^{2}+He_{1}^{2}+En_{2}^{2}+He_{2}^{2}\geq 2\sqrt{\left(En_{1}^{2}+He_{1}^{2}\right)\left(En_{2}^{2}+He_{2}^{2}\right)}$. Hence, $CBD(Y_{T_{1}},Y_{T_{2}})\geq 0$.
(b) Based on Definition 3, if $Y_{T_{1}}=Y_{T_{2}}$, then $Ex_{1}=Ex_{2}$, $En_{1}=En_{2}$, and $He_{1}=He_{2}$. Hence, $CBD(Y_{T_{1}},Y_{T_{2}})=0$.
(c) Obviously provable.

□

### 3.3. Comparison of CBD and WD

In order to demonstrate the effectiveness of the proposed Bhattacharyya distance, this subsection uses CBD and existing Wasserstein distance (WD) to calculate the dissimilarity between given NCMs, respectively, comparing their discrimination abilities through the coefficient of variation (CV). Specifically, employing the data from [20], let two groups of NCMs be $S_{1}=\left\{Y_{T_{1}}=\left(3,3.123,2.05\right),\;Y_{T_{2}}=\left(2,3,1\right),\;Y_{T_{3}}=\left(1.585,3.556,1.358\right)\right\}$ and $S_{2}=\left\{Y_{T_{4}}=\left(1.5,0.62666,0.339\right),\;Y_{T_{5}}=\left(4.6,0.60159,0.30862\right),\;Y_{T_{6}}=\left(4.4,0.75199,0.27676\right),\;Y_{T_{7}}=\left(1.6,0.60159,0.30862\right)\right\}$. The comparison results are shown in Table 3.

From Table 3, it is evident that the results obtained using CBD indicate the greatest dissimilarity between $Y_{T_{1}}$ and $Y_{T_{3}}$, as well as between $Y_{T_{4}}$ and $Y_{T_{5}}$, which is consistent with the results obtained using WD. Furthermore, the CV for CBD is greater than that for WD, implying that CBD can better differentiate NCMs.

**Figure 5:**
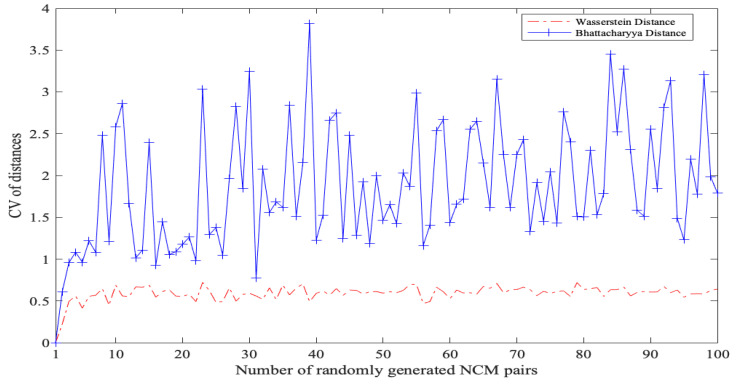
CV of CBD and WD between randomly generated NCMs.

To ensure that the superior performance of CBD is not a special case limited to the given NCMs, we extended our analysis by randomly generating *i* pairs of NCMs (i=1,2,…,100). As shown in Figure 5, the experimental results suggest that the CV of CBD consistently exceeds that of WD, further demonstrating the applicability and effectiveness of the proposed CBD to measure the dissimilarity between NCMs.

## 4. HD-CBDTOPSIS

### 4.1. LSPs’ Evaluation Criteria System

For the purpose of identifying critical evaluation criteria, we explore multiple databases, including Web of Science, CNKI, Google Scholar, Emerald Insight, IEEE Xplore, Elsevier ScienceDirect, Springer Link, Taylor & Francis Online, and Wiley Online Library. The keywords used for the search are supplier evaluation, third-party logistics selection, 3PL selection, cross-border e-commerce logistics, MCDM, etc. Building upon the literature review of 217 papers, this work integrates traditional supplier selection criteria and the cross-border e-commerce transaction process to establish a comprehensive evaluation system and an effective evaluation decision-making method for cross-border LSPs from a risk perspective. The criteria are grouped according to four main aspects, that is, logistics quality, logistics cost, logistics capability, development potential.

A summary of 4 second-level criteria and 37 third-level criteria is provided in Table A1 in Appendix A. However, there may be information redundancy among these indicators. To further screen the final indicators, we refer to [28], which carries out a hypothesis testing on the frequency of each indicator. Our approach differs from [28] in the following ways: (1) Goudarzi and Gholamian [28] performs a two-tailed Z-test, while we conduct a one-tailed Z-test. Since higher frequencies are considered more meaningful, the one-tailed test is more appropriate for scenarios where we focus on whether an indicator’s frequency significantly exceeds the expected frequency. (2) Goudarzi and Gholamian [28] uses the test statistic Z=(p−μ)/σ. However, The frequency’s sampling distribution follows a binomial distribution and is approximately a normal distribution based on the central limit theorem. Therefore, it is more suitable to adopt $Z=\left(p-\mu \right)/\sqrt{\mu (1-\mu )/n}$ as the test statistic. (3) Goudarzi and Gholamian [28] subjectively determines the parameters μ and σ, while we objectively obtain the parameter μ by fitting method.

**Figure 6:**
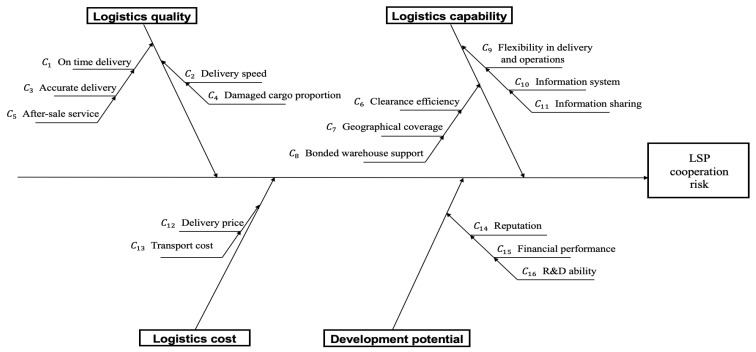
Evaluation criteria system for selecting LSPs.

Assuming that the frequency’s sampling distribution approximately follows a normal distribution $N(\mu ,\sigma^{2})$, it is calculated μ=0.14 by fitted method using maximum likelihood estimation. The null hypothesis and alternative hypothesis are defined as $H_{0}:p\leq \mu ,\;H_{1}:p>\mu$. If the test statistic $Z=\left(p-\mu \right)/\sqrt{\mu (1-\mu )/n}$ is greater than the critical value $Z_{0.05}=1.65$, we have adequate reason to reject $H_{0}$ at the significance level α=0.05. According to the result shown in Table 4, only 12 indicators (marked in black) pass the Z-test. It is worth noting that although certain indicators in the same aspect fail the test individually, they collectively do. Therefore, we merge 9 indicators (dashed annotation) into a new indicator, namely financial performance. Additionally, we combine technical staff proportion and R&D investment ratio into a new indicator, namely R&D ability. Since cross-border e-commerce is an emerging business, the indicators related to its specific processes require time to accumulate for widespread application. Considering the existence bias, we do not perform *Z*-test for clearance efficiency ($C_{3\_1}$) and bonded warehouse support($C_{3\_3})$. Finally, 16 criteria are shortlisted for the further analysis, with the hierarchical structure illustrated in Figure 6.

### 4.2. LSPs’ Evaluation Model

The evaluation and selection of cross-border LSPs, as an MCDM problem, aim to find a compromise solution under conflicting criteria and rank the alternatives from best to worst. Let the criteria set be $C=\{C_{1},C_{2},\ldots ,C_{m}\}$, the alternative LSP set be $A=\{A_{1},A_{2},\ldots ,A_{n}\}$, and the expert set be $DM=\{DM_{1},DM_{2},\ldots ,DM_{p}\}$. The detailed steps of the proposed model—TOPSIS with heterogeneous data and cloud Bhattacharyya distance (*HD-CBDTOPSIS*) are described below.

**Figure 7:**
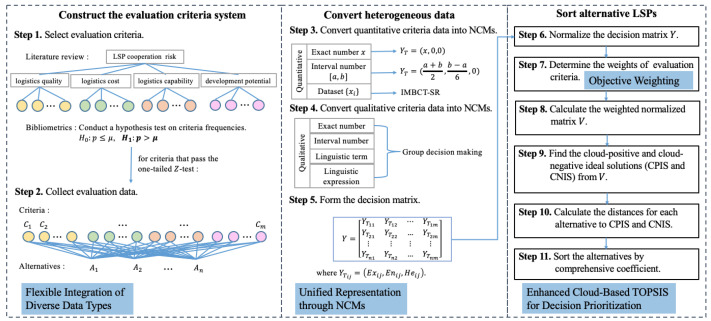
Framework of HD-CBDTOPSIS for selecting LSPs.

HD-CBDTOPSIS consists of 11 steps, and the framework is presented in Figure 7.

The computational complexity of HD-CBDTOPSIS depends on:

(a) The Theta scaling method (Algorithm 3) can convert all linguistic inputs into NCMs before aggregation, which is independent of the complexity of the term sets or the number of experts;
(b) The aggregation of group decision-making (Algorithm 4) do not operate on the original linguistic term sets, but within the NCM feature space, characterized by the values of Ex, En, and He, effectively decoupling the computational complexity from the size of the term set.

**Step 1. Construct the evaluation criteria system.**

As mentioned in Section 4.1, the evaluation criteria system consists of m=16 criteria from four aspects: logistics quality, logistics cost, logistics capability, and development potential.

**Step 2. Collect evaluation data.**

Evaluation data is derived from relevant websites or experts. The data formats could be exact numbers, interval numbers, digital datasets, multi-granularity linguistic terms, or linguistic expressions.

**Step 3. Convert quantitative criteria data into NCMs.**

The data formats for quantitative criteria evaluation could be exact numbers *x*, interval numbers $\left[x_{min},x_{max}\right]$, and digital datasets $\left\{x_{i}\right\}$, which are converted into NCMs in terms of $Y_{T}=\left(x,0,0\right)$, $Y_{T}=\left(\left(x_{min}+x_{max}\right)/2,\left(x_{max}-x_{min}\right)/6,0\right)$, and IMBCT-SR (Algorithm 2), respectively.

**Step 4. Qualitative criteria evaluation based on group decision making and heterogeneous data.**

The heterogeneous data conversion methods for qualitative criteria evaluation are presented in Table 2. Then, utilizing the group decision making method described in Algorithm 4, the qualitative criteria evaluation result is represented by an NCM.

**Step 5. Form the decision matrix represented by NCMs.**

Upon completion of unifying all evaluation data into NCMs, the decision matrix *Y* is

(17) $$Y=\left[Y_{T_{ij}}\right]={\left(\begin{array}{cccc} Y_{T_{11}} & Y_{T_{12}} & \ldots & Y_{T_{1m}}\\ Y_{T_{21}} & Y_{T_{22}} & \ldots & Y_{T_{2m}}\\ \vdots & \vdots & \; & \vdots \\ Y_{T_{n1}} & Y_{T_{n2}} & \ldots & Y_{T_{nm}} \end{array}\right)}_{n\times m}.$$

**Step 6. Normalize the decision matrix.**

For the benefit criteria with higher values being better

(18) $$\gamma_{ij}=\frac{Y_{T_{ij}}-\min_{i}\left(Y_{T_{ij}}\right)}{\max_{i}\left(Y_{T_{ij}}\right)-\min_{i}\left(Y_{T_{ij}}\right)}.$$

For the cost criteria with lower values being better

(19) $$\gamma_{ij}=\frac{\max_{i}\left(Y_{T_{ij}}\right)-Y_{T_{ij}}}{\max_{i}\left(Y_{T_{ij}}\right)-\min_{i}\left(Y_{T_{ij}}\right)}.$$

**Step 7. Determine the weights of evaluation criteria.**

We assign weights objectively based on the variation of each evaluation indicator. If an indicator has a high CV, it indicates that the indicator contains abundant information, allowing clear differentiation among the alternatives. A low CV reflects greater homogeneity across the alternatives, suggesting that this indicator offers limited differentiating utility. Therefore, the indicator with a larger CV should be assigned a high weight, and vice versa.

Calculate the CV of the *j*-th indicator

(20) $$CV_{j}=\frac{SD_{j}}{{\bar{\gamma }}_{j}}=\frac{\sqrt{\frac{1}{n-1}\sum_{i=1}^{n}CBD{\left(\gamma_{ij},{\bar{\gamma }}_{j}\right)}^{2}}}{\frac{1}{n}\sum_{i=1}^{n}\gamma_{ij}},\;j=1,2,\ldots ,m.$$

Calculate the weight of the *j*-th indicator

(21) $$w_{j}=\frac{CV_{j}}{\sum_{j=1}^{m}CV_{j}},\;j=1,2,\ldots ,m.$$

**Step 8. Calculate the weighted normalized matrix $V=[v_{ij}]$.**

(22) $$v_{ij}=w_{j}\times \gamma_{ij}.$$

where $w_{j}$ is the weight of indicator $C_{j}$.

**Step 9. Find the cloud-positive and cloud-negative ideal solutions from *V*.**

The cloud-positive ideal solution (CPIS $Y^{+}$) corresponds to the best score of each indicator, while the cloud-negative ideal solution (CNIS $Y^{-}$) corresponds to the worst score. Both are virtual solutions within the alternatives.

(23) $$\begin{array}{cc} Y^{+} & =\left\{\max_{i}\left(v_{i1}\right),\max_{i}\left(v_{i2}\right),\ldots ,\max_{i}\left(v_{in}\right)\right\}=\left\{Y_{j}^{+}\right\}, \end{array}$$

(24) $$\begin{array}{cc} Y^{-} & =\left\{\min_{i}\left(v_{i1}\right),\min_{i}\left(v_{i2}\right),\ldots ,\min_{i}\left(v_{in}\right)\right\}=\left\{Y_{j}^{-}\right\}. \end{array}$$

**Step 10. Calculate the distances for each alternative to CPIS and CNIS.**

(25) $$\begin{array}{cc} d_{i}^{+} & =\sum_{j=1}^{n}\left(v_{ij}-Y_{j}^{+}\right), \end{array}$$

(26) $$\begin{array}{cc} d_{i}^{-} & =\sum_{j=1}^{n}\left(v_{ij}-Y_{j}^{-}\right). \end{array}$$

**Step 11. Sort the alternatives by comprehensive evaluation coefficient.**

The comprehensive evaluation coefficient $S_{i}$ of each alternative is calculated as

(27) $$S_{i}=\frac{d_{i}^{-}}{d_{i}^{-}+d_{i}^{+}}.$$

The larger the $S_{i}$, the better the alternative. The decision makers can determine the final solution based on the $S_{i}$ in descending order. Notice that $\gamma_{ij}$, $CV_{j}$, $w_{j}$, $v_{ij}$, $d_{i}^{+}$, $d_{i}^{-}$, and $S_{i}$ above are all represented by NCMs. The ranking of alternatives refers to NCMs’ comparison rules (Definition 3).

## 5. A Case Analysis

In this section, we provide an application example of prioritizing cross-border LSPs to demonstrate the feasibility and effectiveness of the proposed *HD-CBDTOPSIS*. The example involves 16 evaluation criteria, with quantitative criteria including on time delivery ($C_{1}$), delivery speed ($C_{2}$), accurate delivery ($C_{3}$), damaged cargo proportion ($C_{4}$), after-sale service ($C_{5}$), clearance efficiency ($C_{6}$), geographical coverage ($C_{7}$), bonded warehouse support ($C_{8}$), delivery price ($C_{12}$), and transport cost ($C_{13}$), and qualitative criteria including flexibility in delivery and operations ($C_{9}$), information system ($C_{10}$), information sharing ($C_{11}$), reputation ($C_{14}$), financial performance ($C_{15}$), and R&D ability ($C_{16}$). There are four alternative LSPs, evaluated by up to seven experts. The evaluation data are listed in Table A2 and Table A3 in Appendix A. The implementation process of the method is described in detail. Finally, the comparative analysis of results is conducted.

### 5.1. Implementation of Proposed Model

The evaluation matrix is shown in Table 5, obtained through conversion methods for heterogeneous data as described in Step 3 and Step 4 in Section 4.2. After normalization, the evaluation matrix is presented in Table 6.

According to Equations (20) and (21), the weights of evaluation criteria are assigned by CV based on CBD, from $C_{1}$ to $C_{16}$ as (0.0323,0.0025,0.0003), (0.2334,0.0411,0.0047), (0.0319,0.0025,0.0003), (0.0122,0.0400,0.0046), (0.0363,0.0028,0.0003), (0.1437,0.0363,0.0013), (0.0451,0.0035,0.0004), (0.0319,0.0025,0.0003), (0.0466,0.0156,0.0026), (0.0920,0.0186,0.0023), (0.0162,0.0151,0.0022), (0.0517,0.0040,0.0005), (0.0489,0.0038,0.0004), (0.0893,0.0242,0.0030), (0.0276,0.0159,0.0022), (0.0608,0.0206,0.0030), respectively. Notice that for exact numbers, CBD cannot be calculated, because En and He are both 0. Therefore, $CBD(\gamma_{ij},{\bar{\gamma }}_{j})$ in Equation (20) is adjusted to $|\gamma_{ij}-{\bar{\gamma }}_{j}|$. The weighted normalized evaluation matrix is shown in Table 7.

Finding the maximum and minimum NCMs for each column, the cloud-positive ideal solution $Y^{+}$ and cloud-negative ideal solution $Y^{-}$ are as follows:

$$\begin{array}{cc} Y^{+}=\{ & (0.0323,0.0025,0.0003),(0.2334,0.0635,0.0076),(0.0319,0.0025,0.0003),(0.0122,0.0590,0.0073),\\ & (0.0363,0.0028,0.0003),(0.1437,0.0423,0.0013),(0.0451,0.0035,0.0004),(0.0319,0.0025,0.0003),\\ & (0.0466,0.0235,0.0037),(0.0920,0.0349,0.0042),(0.0162,0.0226,0.0032),(0.0517,0.0040,0.0005),\\ & (0.0489,0.0038,0.0004),(0.0893,0.0418,0.0047),(0.0276,0.0269,0.0034),(0.0608,0.0421,0.0060)\};\\ Y^{-}=\{ & (0.0000,0.0000,0.0000),(0.0000,0.0216,0.0021),(0.0000,0.0000,0.0000),(0.0000,0.0405,0.0035),\\ & (0.0000,0.0000,0.0000),(0.0000,0.0194,0.0000),(0.0000,0.0000,0.0000),(0.0000,0.0000,0.0000),\\ & (0.0000,0.0137,0.0024),(0.0000,0.0176,0.0025),(0.0000,0.0157,0.0023),(0.0000,0.0000,0.0000),\\ & (0.0000,0.0000,0.0000),(0.0000,0.0138,0.0021),(0.0000,0.0105,0.0018),(0.0000,0.0104,0.0020)\}. \end{array}$$

In addition, the distances for four alternatives to $Y^{+}$ and $Y^{-}$, along with their comprehensive evaluation coefficients, are listed in Table 8. According to NCMs’ comparison rules, the final LSP ranking in descending order is: $A_{3}≻A_{1}≻A_{4}≻A_{2}$. It is evident that $A_{3}$ is the most ideal LSP among the four alternatives.

We further analyze LSPs’ performance on each criterion, as shown in Table 9. According to the normalized evaluation matrix in Table 6, $A_{3}$ performs best in six criteria and ranks in the top two for 10 criteria. Although $A_{2}$ dominates in seven criteria, its bottom-ranking performance in three criteria suggests significant instability. $A_{3}$ surpasses $A_{1}$ and $A_{4}$ on 10 and nine criteria, respectively, demonstrating that $A_{3}$ has better performance than $A_{1}$ and $A_{4}$. $A_{1}$ maximizes scores for $C_{2}$ and $C_{8}$, while $A_{4}$ optimizes $C_{3}$, $C_{4}$, $C_{5}$, $C_{12}$, and $C_{13}$. Considering that $A_{1}$ outperforms $A_{4}$ in 10 criteria, $A_{1}$ should not be inferior to $A_{4}$. These results correspond with the final rankings on the whole. Consequently, $A_{3}$ will be selected as the appropriate LSP for cooperation.

Based on the analyses above, a single alternative is challenging to consistently guarantee superiority across all criteria. Therefore, it is valuable to develop an MCDM approach to evaluate alternatives and identify the potential optimal alternative. The proposed HD-CBDTOPSIS considers both quantitative and qualitative criteria, involves heterogeneous data and group decision-making, and represents evaluation results as NCMs to preserve uncertainty, making the LSP selection more scientific and reliable.

### 5.2. Comparative Analysis

Numerous studies about MCDM problems have employed one or more of the following methods: fuzzy set theory, AHP, grey relational analysis, cloud model, VIKOR, or TOPSIS. To the authors’ best knowledge, few studies have simultaneously considered quantitative and qualitative criteria involving heterogeneous data for supplier selection. Our work is the first to deal with the most comprehensive data types. Given that previous methods have not achieved the same condition as our proposed model, a quantitative comparison is meaningless and unfair. Therefore, we conduct a qualitative comparative analysis to demonstrate the substantial advantages of HD-CBDTOPSIS, comparing it with six recently published methods, as listed in Table 10.

Basic statistical calculations in [43] result in the loss of evaluation information, making it difficult to reflect the essence from complex data. In [6,17,44,45,46,47], fuzzy sets and their extensions are used to illustrate the uncertainty of original data. HD-CBDTOPSIS, based on probability theory and fuzzy sets known as NCMs, possesses a more rigorous mathematical foundation. The NCMs describe the degree of fuzziness and randomness more precisely through three numerical characteristics, thereby offering more reasonable solutions to practical problems. The subjective weight assignment to criteria in [6,44,46,47,48,49] is inevitably influenced by experts’ preferences, which introduces uncertainty into the evaluation results. The subjective weighting methods based on pairwise comparisons, such as AHP [46] and BWM [44], require complex cognitive calculations. These approaches lose flexibility when dealing with case studies with a large number of criteria. Both entropy weight [17] and CV are typical objective weighting methods, reasonably reflecting criteria importance from the data characteristics. In addition, HD-CBDTOPSIS also considers the quality of decision information to determine the experts’ weights in group decision-making for qualitative criteria evaluation. The traditional ranking algorithms, such as grey relational analysis [45,46], TOPSIS [6,17,43,47,49], or VIKOR [43], provide final rankings as definitive values that could not reflect uncertainty in the results. In contrast, our proposed HD-CBDTOPSIS retains uncertainty throughout the evaluation process.

**Table 10:** Comparative analysis with the latest methods.

Method	Data Type	Data Conversion	Weight Determination	Ranking
Hendiani and Walther [6]	Linguistic terms	Interval intuitionistic fuzzy set	Subjective weight	TOPSIS
Dorfeshan et al. [44]	Linguistic terms	Triangular interval fuzzy soft sets	BWM	TOPSIS
Wang et al. [45]	Linguistic terms	Interval type-2 fuzzy set	AHP and entropy weight	Grey MABAC
Bai and Sarkis [43]	Exact numbers Linguistic terms	Quantitative criteria: statistical calculation Qualitative criteria: numerical scale table, statistical calculation	/	Neighborhood rough set and TOPSIS-VIKOR
Zarbakhshnia et al. [46]	Linguistic terms	Triangular fuzzy number	AHP	MOORA-G
Chen et al. [48]	Linguistic expressions	Hesitant fuzzy linguistic term set probability distribution, group decision making	Subjective weight, Triangular fuzzy number	Expectation of probability distributions
Li et al. [17]	Exact numbers	Generalized fuzzy number, group decision making	Entropy weight	Fuzzy TOPSIS
Su et al. [47]	Interval numbers	Interval intuitionistic fuzzy set, group decision making	Subjective weight	TOPSIS
Jadidi et al. [49]	Exact numbers	/	Subjective weight	TOPSIS
Proposed HD-CBDTOPSIS	Exact numbers, Interval numbers, Digital datasets, Multi-granularity linguistic terms, Linguistic expressions	Quantitative criteria: statistical calculation and IMBCT-SR Qualitative criteria: group decision making	CV based on CBD	Cloud TOPSIS

## 6. Conclusions

In this paper, we propose a hybrid multi-criteria group decision-making (MCGDM) model, termed **HD-CBDTOPSIS**, which integrates heterogeneous data, group decision-making mechanisms, the cloud model, and the TOPSIS method to rank cross-border logistics service providers (LSPs). By combining traditional supplier selection criteria with key aspects of cross-border e-commerce transactions, we construct a comprehensive evaluation system encompassing both quantitative and qualitative indicators. The model accommodates a wide range of data formats, including precise numerical values, interval numbers, digital datasets, multi-granularity linguistic terms, and linguistic expressions. To the best of our knowledge, no existing work has considered such a rich variety of heterogeneous data within a unified framework.

HD-CBDTOPSIS addresses several limitations of existing MCDM models and makes the following key contributions:

- A set of evaluation methods based on normal cloud models (NCMs) is developed. In particular, the IMBCT-SR algorithm is introduced for handling quantitative criteria represented by digital datasets. For qualitative indicators, a novel group decision-making approach is proposed to effectively handle diverse linguistic inputs.
- An objective weighting mechanism is adopted, where criterion weights are determined using the coefficient of variation (CV) in conjunction with the cloud-based dissimilarity (CBD) metric. The CBD is specifically designed to measure differences between NCMs and has demonstrated superior discriminatory power in our experiments.
- A cloud-based TOPSIS method is employed to rank the alternatives, ensuring that uncertainty is preserved to the greatest extent possible.
- The feasibility, effectiveness, and flexibility of the HD-CBDTOPSIS model are validated through an illustrative application and comparative analysis.

This method provides cross-border e-commerce administrators with a robust, data-driven framework for supplier management. It moves beyond traditional performance metrics by integrating both quantitative and qualitative factors, providing a more nuanced understanding of logistics services. This holistic perspective enables administrators to optimize decisions in partnership development, resource allocation, and supply chain configuration, ensuring improved strategic alignment between logistics capabilities and business objectives. By adopting this method, enterprises can cultivate resilient supply networks, improve customer satisfaction through reliable fulfillment, and ultimately strengthen their marketplace position.

Despite its advantages, the model also has certain limitations, which point to future research directions. First, the evaluation framework can be expanded by incorporating additional external factors, such as tariffs, exchange rates, and product-specific attributes, thereby enriching the informational dimensions of the decision-making process. Second, the current model does not account for potential interdependencies among criteria. To build a more robust and scientifically grounded approach, future research could integrate HD-CBDTOPSIS with methods such as grey relational analysis and DEMATEL. Third, our proposed HD-CBDTOPSIS relies on three implicit assumptions: (1) the chosen linguistic term sets (e.g., 5-term, 7-term, and 9-term) adequately capture experts’ semantic space; (2) the linguistic term granularity remains consistent for individual expert during a single evaluation of a given candidate; and (3) the degree of uncertainty and consistency are treated equally, i.e., α=0.5 in Equation (12). If scenarios in which experts disagree strongly are taken into account, it may be necessary to dynamically expand a more granular linguistic term set (e.g., from 7-term to 15-term) to distinguish subtle differences. To further enrich the expression methods of linguistic information, in future research, the modeling of hierarchical linguistic terms and probabilistic linguistic terms can be considered to enhance the flexibility and diversity of experts in expressing qualitative viewpoints. Moreover, developing a hybrid weighting scheme that combines subjective and objective perspectives—such as the integrated approach proposed by Wang et al. [45]—would further enhance the model’s applicability. Finally, HD-CBDTOPSIS shows strong potential for addressing large-scale MCGDM problems and can be extended to empirical studies in domains beyond LSP selection.

## Figures and Tables

**Table 1:** Seven linguistic terms encoded into NCM.

Linguistic Terms	θ	NCMs
$T_{-3}:\;$none	0	(0,2.9650,0.1228)
$T_{-2}:\;$very low (vl)	0.2210	(2.2097,2.6631,0.2234)
$T_{-1}:\;$low	0.3823	(3.8227,2.1075,0.4086)
$T_{0}\;:\;$medium (m)	0.5000	(5,1.9283,0.4683)
$T_{1}\;:\;$high	0.6177	(6.1773,2.1075,0.4086)
$T_{2}\;:\;$very high (vh)	0.7790	(7.7903,2.6631,0.2234)
$T_{3}\;:\;$perfect	1	(10,2.9650,0.1228)

**Table 2:** Heterogeneous data conversion to NCM.

Data Format	NCM
exact number x	$Y_{T}=\left(x,0,0\right)$
interval number $\;[x_{min},x_{max}]$	$Y_{T}=\left(\frac{x_{min}+x_{max}}{2},\frac{x_{max}-x_{min}}{6},0\right)$
linguistic term	The theta scaling method (Algorithm 3)
linguistic expression	(1) HCLTS mapping: $T_{ll}\overset{\mathrm{Definition}\;9}{\to }H_{T}$ (2) Synthetic operation: $H_{T}\overset{\;\mathrm{Definition}\;4}{\to }Y_{T}$

**Table 3:** CBD and WD between given NCMs.

Distance	Group $S_{1}$			Group $S_{2}$						CV
	$<Y_{T_{1}},Y_{T_{2}}>$	$<Y_{T_{1}},Y_{T_{3}}>$	$<Y_{T_{2}},Y_{T_{3}}>$	$<Y_{T_{4}},Y_{T_{5}}>$	$<Y_{T_{4}},Y_{T_{6}}>$	$<Y_{T_{4}},Y_{T_{7}}>$	$<Y_{T_{5}},Y_{T_{6}}>$	$<Y_{T_{5}},Y_{T_{7}}>$	$<Y_{T_{6}},Y_{T_{7}}>$	
WD	1.1528	1.4168	0.7663	3.1002	2.9014	0.1064	0.2359	3.0000	2.8028	0.7191
CBD	0.0173	0.0177	0.0103	2.4909	1.8322	0.0033	0.0163	2.4609	1.7902	1.1952

**Table 4:** Criteria screening by Z-test. Here, * stands for p<0.05 and ** stands for p<0.01, respectively.

Indicator	Symbol	Frequency	*n*	*p*	μ	SE	95% CI	Δ	Var	*Z*	*p*-Value
				Frequency/n		$\sqrt{p(1-p)/n}$	[p−$Z_{\alpha }\times$SE,p+$Z_{\alpha }\times$SE]	p−μ	$\sqrt{\mu (1-\mu )/n}$	Δ/Var	
**On time delivery**	$C_{1\_1}$	90	217	0.4147	0.14	0.0334	[0.3596, 0.4699]	0.2747	0.0236	11.6640	0.0000 **
**Delivery speed**	$C_{1\_2}$	64	217	0.2949	0.14	0.0310	[0.2439, 0.3460]	0.1549	0.0236	6.5774	0.0000 **
**Accurate delivery**	$C_{1\_3}$	46	217	0.2120	0.14	0.0277	[0.1662, 0.2578]	0.0720	0.0236	3.0559	0.0011 *
**Damaged cargo proportion**	$C_{1\_4}$	62	217	0.2857	0.14	0.0307	[0.2351, 0.3363]	0.1457	0.0236	6.1861	0.0000 **
Customer satisfaction	$C_{1\_5}$	35	217	0.1613	0.14	0.0250	[0.1201, 0.2025]	0.0213	0.0236	0.9039	0.1830
**After-sale service**	$C_{1\_6}$	39	217	0.1797	0.14	0.0261	[0.1367, 0.2227]	0.0397	0.0236	1.6864	0.0459 *
**Delivery price**	$C_{2\_1}$	59	217	0.2719	0.14	0.0302	[0.2221, 0.3217]	0.1319	0.0236	5.5992	0.0000 **
**Transport cost**	$C_{2\_2}$	47	217	0.2166	0.14	0.0280	[0.1705, 0.2627]	0.0766	0.0236	3.2515	0.0006 **
Storage cost	$C_{2\_3}$	25	217	0.1152	0.14	0.0217	[0.0794, 0.1510]	−0.0248	0.0236	−1.0525	0.8537
Packing level	$C_{2\_4}$	18	217	0.0829	0.14	0.0187	[0.0521, 0.1138]	−0.0571	0.0236	−2.4220	0.9923
Settlement cycle	$C_{2\_5}$	6	217	0.0276	0.14	0.0111	[0.0093, 0.0460]	−0.1124	0.0236	−4.7697	1.0000
**Geographical coverage**	$C_{3\_2}$	42	217	0.1935	0.14	0.0268	[0.1493, 0.2378]	0.0535	0.0236	2.2733	0.0115 *
**Flexibility** **in delivery and operations**	$C_{3\_4}$	75	217	0.3456	0.14	0.0323	[0.2924, 0.3989]	0.2056	0.0236	8.7294	0.0000 **
Communication	$C_{3\_5}$	29	217	0.1336	0.14	0.0231	[0.0955, 0.1718]	−0.0064	0.0236	−0.2700	0.6064
**Information system**	$C_{3\_6}$	105	217	0.4839	0.14	0.0339	[0.4279, 0.5398]	0.3439	0.0236	14.5986	0.0000 **
**Information sharing**	$C_{3\_7}$	45	217	0.2074	0.14	0.0275	[0.1620, 0.2528]	0.0674	0.0236	2.8603	0.0021 **
Brand operating time	$C_{4\_1}$	6	217	0.0276	0.14	0.0111	[0.0093, 0.0460]	−0.1124	0.0236	−4.7697	1.0000
Number of employees	$C_{4\_2}$	18	217	0.0829	0.14	0.0187	[0.0521, 0.1138]	−0.0571	0.0236	−2.4220	0.9923
Managerial staff proportion	$C_{4\_3}$	5	217	0.0230	0.14	0.0102	[0.0062, 0.0398]	−0.1170	0.0236	−4.9653	1.0000
Employee turnover rate	$C_{4\_5}$	9	217	0.0415	0.14	0.0135	[0.0191, 0.0638]	−0.0985	0.0236	−4.1828	1.0000
Historical partnership	$C_{4\_6}$	4	217	0.0184	0.14	0.0091	[0.0034, 0.0335]	−0.1216	0.0236	−5.1610	1.0000
Cooperation Duration	$C_{4\_7}$	2	217	0.0092	0.14	0.0065	[−0.0015, 0.0199]	−0.1308	0.0236	−5.5523	1.0000
Trust	$C_{4\_8}$	25	217	0.1152	0.14	0.0217	[0.0794, 0.1510]	−0.0248	0.0236	−1.0525	0.8537
**Reputation**	$C_{4\_9}$	55	217	0.2535	0.14	0.0295	[0.2047, 0.3022]	0.1135	0.0236	4.8166	0.0000 **
**Financial performance**		66	217	0.3041	0.14	0.0312	[0.2526, 0.3557]	0.1641	0.0236	6.9687	0.0000 **
Market share	$C_{4\_10}$	18	217	0.0829	0.14	0.0187	[0.0521, 0.1138]	−0.0571	0.0236	−2.4220	0.9923
Revenue	$C_{4\_11}$	13	217	0.0599	0.14	0.0161	[0.0333, 0.0865]	−0.0801	0.0236	−3.4002	0.9997
Revenue growth rate	$C_{4\_12}$	7	217	0.0323	0.14	0.0120	[0.0125, 0.0520]	−0.1077	0.0236	−4.5741	1.0000
Return on equity (ROE)	$C_{4\_13}$	8	217	0.0369	0.14	0.0128	[0.0158, 0.0580]	−0.1031	0.0236	−4.3784	1.0000
Return on investment (ROI)	$C_{4\_14}$	7	217	0.0323	0.14	0.0120	[0.0125, 0.0520]	−0.1077	0.0236	−4.5741	1.0000
Investment in fixed assets	$C_{4\_15}$	32	217	0.1475	0.14	0.0241	[0.1078, 0.1872]	0.0075	0.0236	0.3169	0.3756
Investment growth rate in fixed assets	$C_{4\_16}$	2	217	0.0092	0.14	0.0065	[−0.0015, 0.0199]	−0.1308	0.0236	−5.5523	1.0000
Accounts receivable turnover ratio	$C_{4\_18}$	2	217	0.0092	0.14	0.0065	[−0.0015, 0.0199]	−0.1308	0.0236	−5.5523	1.0000
Asset liability ratio	$C_{4\_19}$	8	217	0.0369	0.14	0.0128	[0.0158, 0.0580]	−0.1031	0.0236	−4.3784	1.0000
**R&D ability**		50	217	0.2304	0.14	0.0286	[0.1832, 0.2776]	0.0904	0.0236	3.8385	0.0001 **
Technical staff proportion	$C_{4\_4}$	29	217	0.1336	0.14	0.0231	[0.0955, 0.1718]	−0.0064	0.0236	−0.2700	0.6064
R&D investment ratio	$C_{4\_17}$	30	217	0.1382	0.14	0.0234	[0.0996, 0.1769]	−0.0018	0.0236	−0.0743	0.5296

**Table 5:** The evaluation matrix of 4 alternative LSPs on 16 criteria.

	$C_{1}$	$C_{2}$	$C_{3}$	$C_{4}$	$C_{5}$	$C_{6}$	$C_{7}$	$C_{8}$
$A_{1}$	(0.9300, 0.0000, 0.0000)	(35.9962, 1.9638, 0.2521)	(0.9800, 0.0000, 0.0000)	(2.0197, 3.1173, 0.2692)	(5.0000, 0.0000, 0.0000)	(3.5000, 0.5000, 0.0000)	(50,000, 0.0000, 0.0000)	(1.0000, 0.0000, 0.0000)
$A_{2}$	(0.9600, 0.0000, 0.0000)	(50.9823, 0.9784, 0.0951)	(1.0000, 0.0000, 0.0000)	(1.7725, 3.0052, 0.2727)	(2.0000, 0.0000, 0.0000)	(6.0000, 0.3333, 0.0000)	(227,000, 0.0000, 0.0000)	(1.0000, 0.0000, 0.0000)
$A_{3}$	(1.0000, 0.0000, 0.0000)	(39.9603, 0.5688, 0.0537)	(1.0000, 0.0000, 0.0000)	(0.8316, 1.5847, 0.3990)	(3.0000, 0.0000, 0.0000)	(2.5000, 0.1667, 0.0000)	(170,000, 0.0000, 0.0000)	(1.0000, 0.0000, 0.0000)
$A_{4}$	(0.6000, 0.0000, 0.0000)	(36.4410, 4.4900, 0.5088)	(1.0000, 0.0000, 0.0000)	(0.6883, 1.1815, 0.3484)	(1.0000, 0.0000, 0.0000)	(5.0000, 1.3333, 0.0000)	(90,000, 0.0000, 0.0000)	(0.0000, 0.0000, 0.0000)
	$C_{9}$	$C_{10}$	$C_{11}$	$C_{12}$	$C_{13}$	$C_{14}$	$C_{15}$	$C_{16}$
$A_{1}$	(7.1027, 0.6331, 0.1259)	(8.3838, 0.6540, 0.0836)	(7.0391, 0.7855, 0.1269)	(1.1500, 0.0000, 0.0000)	(50.0000, 0.0000, 0.0000)	(7.0417, 0.8211, 0.1085)	(7.9031, 0.9565, 0.1362)	(7.3638, 0.7480, 0.1272)
$A_{2}$	(8.4497, 0.5390, 0.0632)	(8.7204, 1.0231, 0.1045)	(8.5056, 0.5344, 0.0494)	(1.1000, 0.0000, 0.0000)	(45.0000, 0.0000, 0.0000)	(7.2888, 0.4961, 0.1052)	(9.5307, 1.7217, 0.1661)	(3.7957, 0.4328, 0.0816)
$A_{3}$	(5.2603, 0.6626, 0.1149)	(8.3247, 0.4822, 0.0451)	(8.0011, 0.6853, 0.0865)	(1.0500, 0.0000, 0.0000)	(48.0000, 0.0000, 0.0000)	(9.0135, 0.9922, 0.0942)	(5.9762, 0.9568, 0.1660)	(7.3741, 1.4650, 0.1988)
$A_{4}$	(6.5076, 0.4496, 0.0829)	(3.1036, 0.7606, 0.1096)	(6.4887, 1.3772, 0.2015)	(0.9000, 0.0000, 0.0000)	(40.0000, 0.0000, 0.0000)	(4.9817, 0.4418, 0.0669)	(7.0548, 0.5204, 0.0448)	(6.0507, 0.6020, 0.1167)

**Table 6:** The normalized evaluation matrix of 4 alternative LSPs on 16 criteria.

	$C_{1}$	$C_{2}$	$C_{3}$	$C_{4}$	$C_{5}$	$C_{6}$	$C_{7}$	$C_{8}$
$A_{1}$	(0.8250, 0.0000, 0.0000)	(1.0000, 0.2070, 0.0254)	(0.0000, 0.0000, 0.0000)	(0.0000, 3.3112, 0.2859)	(0.0000, 0.0000, 0.0000)	(0.7143, 0.1878, 0.0000)	(0.0000, 0.0000, 0.0000)	(1.0000, 0.0000, 0.0000)
$A_{2}$	(0.9000, 0.0000, 0.0000)	(0.0000, 0.0923, 0.0090)	(1.0000, 0.0000, 0.0000)	(0.1857, 3.2853, 0.2943)	(0.7500, 0.0000, 0.0000)	(0.0000, 0.1347, 0.0000)	(1.0000, 0.0000, 0.0000)	(1.0000, 0.0000, 0.0000)
$A_{3}$	(1.0000, 0.0000, 0.0000)	(0.7355, 0.1315, 0.0151)	(1.0000, 0.0000, 0.0000)	(0.8924, 3.4484, 0.4667)	(0.5000, 0.0000, 0.0000)	(1.0000, 0.1506, 0.0000)	(0.6780, 0.0000, 0.0000)	(1.0000, 0.0000, 0.0000)
$A_{4}$	(0.0000, 0.0000, 0.0000)	(0.9703, 0.3379, 0.0387)	(1.0000, 0.0000, 0.0000)	(1.0000, 3.5410, 0.4677)	(1.0000, 0.0000, 0.0000)	(0.2857, 0.3938, 0.0000)	(0.2260, 0.0000, 0.0000)	(0.0000, 0.0000, 0.0000)
	$C_{9}$	$C_{10}$	$C_{11}$	$C_{12}$	$C_{13}$	$C_{14}$	$C_{15}$	$C_{16}$
$A_{1}$	(0.5777, 0.3263, 0.0585)	(0.9401, 0.2782, 0.0353)	(0.2729, 0.8111, 0.1214)	(0.0000, 0.0000, 0.0000)	(0.0000, 0.0000, 0.0000)	(0.5109, 0.2691, 0.0348)	(0.5421, 0.4849, 0.0702)	(0.9971, 0.4894, 0.0733)
$A_{2}$	(1.0000, 0.3787, 0.0581)	(1.0000, 0.3210, 0.0381)	(1.0000, 1.0358, 0.1455)	(0.2000, 0.0000, 0.0000)	(0.5000, 0.0000, 0.0000)	(0.5722, 0.2256, 0.0350)	(1.0000, 0.7837, 0.0934)	(0.0000, 0.1710, 0.0322)
$A_{3}$	(0.0000, 0.2938, 0.0509)	(0.9296, 0.2650, 0.0328)	(0.7499, 0.9399, 0.1333)	(0.4000, 0.0000, 0.0000)	(0.2000, 0.0000, 0.0000)	(1.0000, 0.3810, 0.0405)	(0.0000, 0.3807, 0.0660)	(1.0000, 0.6037, 0.0849)
$A_{4}$	(0.3911, 0.2720, 0.0472)	(0.0000, 0.1915, 0.0276)	(0.0000, 0.9657, 0.1413)	(1.0000, 0.0000, 0.0000)	(1.0000, 0.0000, 0.0000)	(0.0000, 0.1550, 0.0235)	(0.3034, 0.3495, 0.0524)	(0.6302, 0.3396, 0.0549)

**Table 7:** The weighted normalized evaluation matrix of 4 alternative LSPs on 16 criteria.

	$C_{1}$	$C_{2}$	$C_{3}$	$C_{4}$	$C_{5}$	$C_{6}$	$C_{7}$	$C_{8}$
$A_{1}$	(0.0266, 0.0021, 0.0002)	(0.2334, 0.0635, 0.0076)	(0.0000, 0.0000, 0.0000)	(0.0000, 0.0405, 0.0035)	(0.0000, 0.0000, 0.0000)	(0.1027, 0.0374, 0.0009)	(0.0000, 0.0000, 0.0000)	(0.0319, 0.0025, 0.0003)
$A_{2}$	(0.0291, 0.0023, 0.0003)	(0.0000, 0.0216, 0.0021)	(0.0319, 0.0025, 0.0003)	(0.0023, 0.0409, 0.0037)	(0.0272, 0.0021, 0.0002)	(0.0000, 0.0194, 0.0000)	(0.0451, 0.0035, 0.0004)	(0.0319, 0.0025, 0.0003)
$A_{3}$	(0.0323, 0.0025, 0.0003)	(0.1717, 0.0431, 0.0050)	(0.0319, 0.0025, 0.0003)	(0.0109, 0.0553, 0.0070)	(0.0182, 0.0014, 0.0002)	(0.1437, 0.0432, 0.0013)	(0.0306, 0.0024, 0.0003)	(0.0319, 0.0025, 0.0003)
$A_{4}$	(0.0000, 0.0000, 0.0000)	(0.2265, 0.0884, 0.0101)	(0.0319, 0.0025, 0.0003)	(0.0122, 0.0590, 0.0073)	(0.0363, 0.0028, 0.0003)	(0.0411, 0.0576, 0.0004)	(0.0102, 0.0008, 0.0001)	(0.0000, 0.0000, 0.0000)
	$C_{9}$	$C_{10}$	$C_{11}$	$C_{12}$	$C_{13}$	$C_{14}$	$C_{15}$	$C_{16}$
$A_{1}$	(0.0269, 0.0177, 0.0031)	(0.0865, 0.0310, 0.0039)	(0.0044, 0.0138, 0.0021)	(0.0000, 0.0000, 0.0000)	(0.0000, 0.0000, 0.0000)	(0.0456, 0.0270, 0.0035)	(0.0150, 0.0159, 0.0023)	(0.0607, 0.0362, 0.0054)
$A_{2}$	(0.0466, 0.0235, 0.0037)	(0.0920, 0.0349, 0.0042)	(0.0162, 0.0226, 0.0032)	(0.0103, 0.0008, 0.0001)	(0.0245, 0.0019, 0.0002)	(0.0511, 0.0245, 0.0936)	(0.0276, 0.0269, 0.0034)	(0.0000, 0.0104, 0.0020)
$A_{3}$	(0.0000, 0.0137, 0.0024)	(0.0855, 0.0299, 0.0037)	(0.0122, 0.0190, 0.0027)	(0.0207, 0.0016, 0.0002)	(0.0098, 0.0008, 0.0001)	(0.0893, 0.0418, 0.0047)	(0.0000, 0.0105, 0.0018)	(0.0608, 0.0421, 0.0060)
$A_{4}$	(0.0182, 0.0141, 0.0024)	(0.0000, 0.0176, 0.0025)	(0.0000, 0.0157, 0.0023)	(0.0517, 0.0040, 0.0005)	(0.0489, 0.0038, 0.0004)	(0.0000, 0.0138, 0.0021)	(0.0084, 0.0108, 0.0016)	(0.0383, 0.0244, 0.0038)

**Table 8:** The rankings of 4 alternative LSPs.

Alternatives	Distance to $Y^{+}$	Distance to $Y^{-}$	Comprehensive Evaluation Coefficient	Ranking
$A_{1}$	(0.3663,0.1635,0.0193)	(0.6337,0.1203,0.0138)	(0.6337,0.1761,0.0204)	2
$A_{2}$	(0.5643,0.1488,0.0177)	(0.4357,0.0995,0.0116)	(0.4357,0.1264,0.0148)	4
$A_{3}$	(0.2506,0.1664,0.0197)	(0.7494,0.1242,0.0145)	(0.7494,0.1991,0.0234)	1
$A_{4}$	(0.4763,0.1794,0.0205)	(0.5237,0.1412,0.0156)	(0.5237,0.1850,0.0206)	3

**Table 9:** The rankings of 4 alternative LSPs on each criterion.

Criteria	Ranking	Criteria	Ranking
$C_{1}$	$A_{3}≻A_{2}≻A_{1}≻A_{4}$	$C_{9}$	$A_{2}≻A_{1}≻A_{4}≻A_{3}$
$C_{2}$	$A_{1}≻A_{4}≻A_{3}≻A_{2}$	$C_{10}$	$A_{2}≻A_{1}≻A_{3}≻A_{4}$
$C_{3}$	$A_{2}∼A_{3}∼A_{4}≻A_{1}$	$C_{11}$	$A_{2}≻A_{3}≻A_{1}≻A_{4}$
$C_{4}$	$A_{4}≻A_{3}≻A_{2}≻A_{1}$	$C_{12}$	$A_{4}≻A_{3}≻A_{2}≻A_{1}$
$C_{5}$	$A_{4}≻A_{2}≻A_{3}≻A_{1}$	$C_{13}$	$A_{4}≻A_{2}≻A_{3}≻A_{1}$
$C_{6}$	$A_{3}≻A_{1}≻A_{4}≻A_{2}$	$C_{14}$	$A_{3}≻A_{2}≻A_{1}≻A_{4}$
$C_{7}$	$A_{2}≻A_{3}≻A_{4}≻A_{1}$	$C_{15}$	$A_{2}≻A_{1}≻A_{4}≻A_{3}$
$C_{8}$	$A_{1}∼A_{2}∼A_{3}≻A_{4}$	$C_{16}$	$A_{3}≻A_{1}≻A_{4}≻A_{2}$

## Data Availability

The source code and data presented in this study are available in Appendix A and https://pan.baidu.com/s/1lY6tHWLQi0Zmj_QwTZU-og?pwd=g3tj (accessed on 17 August 2025).

## Appendix A

**Table A1:** Risk evaluation criteria for LSPs in cross-border e-commerce.

Criteria		Sub-Criteria		Indicator				Attribute	Direction	Main Sources
Variable	Symbol	Variable	Symbol	Variable	Symbol	Description	Measure			
Cooperation risk	CR	Logistics quality	$C_{1}$	On time delivery	$C_{1\_1}$	Logistics quality is typically assessed from four perspectives: delivery punctuality, timeliness, accuracy and security. Punctuality $\left(C_{1\_1}\right)$ refers to the ability of LSPs to deliver orders to consumers as promised.	on time delivery/total shipments	Quantitative	+	[12,50,51,52,53,54,55,56]
				Delivery speed	$C_{1\_2}$	Timeliness $\left(C_{1\_2}\right)$ refers to the transportation speed of LSPs, namely the time from shipping origin to destination	{ lead time in order *i* } (i=1,…,N)	Quantitative	–	[46,50,51,57,58]
				Accurate delivery	$C_{1\_3}$	Accuracy $\left(C_{1\_3}\right)$ refers to the degree to which the cargoes actually delivered match the orders, including the types, models and quantities.	accurate delivery/total shipments	Quantitative	+	[57,59]
				Damaged cargo proportion	$C_{1\_4}$	Security $\left(C_{1\_4}\right)$ refers to the ability of LSPs to ensure that cargoes are not damaged or lost during transportation, loading and unloading.	{ damaged cargo percentage in order *i* } (i=1,…,N)	Quantitative	–	[8,12,53,59,60,61]
				Customer satisfaction	$C_{1\_5}$	Satisfaction $\left(C_{1\_5}\right)$ represents customer feedback on the logistics quality.	consumer rating: 1 to 5 stars	Qualitative	+	[2,8,56,59]
				After-sale service	$C_{1\_6}$	After-sales service $\left(C_{1\_6}\right)$ refers to the efficiency of LSPs in handling complaints such as claims and returns. Considering the gap in culture and distance, processing international returns is more complex than domestic returns. The indicator reflects LSPs’ reverse logistics ability.	$\frac{1}{N}\sum_{i=1}^{N}\left(CRT_{i}\right)$ CRT: complaint resolution time	Quantitative	–	[55,57,62]
		Logistics cost	$C_{2}$	Delivery price	$C_{2\_1}$	The LSP price per tonne-kilometer compared with the industry average price reflects the rationality of charges.	LSP’s quoted price/industry average price	Quantitative	–	[16,63]
				Transport cost	$C_{2\_2}$	Logistics cost is affected by transport mode, commodity properties, order properties, value-added services, market fluctuation, etc. Transport cost is the most significant part in the total cost, consisting of the cost of transporting cargoes internationally and domestically. Storage cost is the sum of the factors invested in warehousing related activities, such as rent, human resources, energy and equipment maintenance. Packaging level refers to the ability of LSPs to appropriately package cargoes according to product characteristics and customs standards.	industry report, questionnaire	Quantitative	–	[56,59,61,64]
				Storage cost	$C_{2\_3}$		industry report, questionnaire	Quantitative	–	[56,59,61,64]
				Packing level	$C_{2\_4}$		questionnaire	Qualitative	+	[8,56,62]
				Settlement cycle	$C_{2\_5}$	$Y_{2\_5}$ is defined as the period for the LSP to complete transportation and receive payment from the e-commerce enterprise. The longer the period, the greater the benefit to the enterprise.	questionnaire	Quantitative	+	[54,55]
		Logistics capability	$C_{3}$	Clearance efficiency	$C_{3\_1}$	Clearance efficiency directly affects lead time and cost control, which is one of the core competences of cross-border LSPs. Stable and fast clearance requires LSPs to be familiar with policies of importing and exporting countries, to ensure documents correctness and completeness, and to comply with packing standards for special cargoes.	estimated clearance time	Quantitative	–	[4,57,60,65,66]
				Geographical coverage	$C_{3\_2}$	Wider geographical coverage creates access to capture market share.	number of operational hubs	Quantitative	+	[12,57,67,68,69]
				Bonded warehouse support	$C_{3\_3}$	LSPs may allow their clients to take advantage of bonded warehouses, facilitating cost saving and clearance acceleration.	questionnaire	Quantitative	+	[57]
				Flexibility in delivery and operations	$C_{3\_4}$	It refers to the ability to adapt to changing and unforeseen circumstances, such as urgent requirements or customized services.	questionnaire	Qualitative	+	[8,12,46,68]
				Communication	$C_{3\_5}$	Effective communication refers to unblocked channels, attitude of service staff, responding and understanding ability of requirements.	questionnaire	Qualitative	+	[54,55,57,62,70,71]
				Information system	$C_{3\_6}$	It is related to digitization level, including information accessibility and security on computer networks, adoption of EDI, ERP, WMS, GPS, GIS, TMS, tracking/tracing technologies, etc.	questionnaire	Qualitative	+	[7,8,54,62,68,69,72,73,74]
				Information sharing	$C_{3\_7}$	Partners in the supply chain can reduce the bullwhip effect and make better decisions by information sharing. $C_{3\_7}$ refers to the willingness of partners to share right market information.	questionnaire	Qualitative	+	[7,55,70,72]
		Development potential	$C_{4}$	Brand operating time	$C_{4\_1}$	LSPs’ scale is described by its operating time and employee composition. A long operating history and large employee base both reflect rich logistics experience and mature logistics capabilities.	current date − incorporation date	Quantitative	+	[4,74,75]
						Number of employees	$C_{4\_2}$		questionnaire	Quantitative	+	[59,61]
						Managerial staff proportion	$C_{4\_3}$	Managerial staff typically includes top management members, team leaders, and professional consultants, such as legal and financial advisors.	the percentage of managerial staff	Quantitative	+	[73,75]
						Technical staff proportion	$C_{4\_4}$	Technical staff that has formal technical training include IT engineers, data analysts, transportation employees, especially those with qualification to transport dangerous or perishable cargoes.	the percentage of technical staff	Quantitative	+	[2,16,53,73]
						Employee turnover rate	$C_{4\_5}$	The low turnover rate of technical teams and front-line transport employees ensures the stability of logistics quality.	the percentage of employees resigning voluntarily in a year	Quantitative	–	[56]
						Historical partnership	$C_{4\_6}$	The evaluations of an LSP by historical partners are true reflections of their capabilities. Cooperating with LSPs with higher evaluations will reduce cooperation risk.	amount of companies with 5+ years of cooperation	Quantitative	+	[56,58,74]
						Cooperation Duration	$C_{4\_7}$	Long-term partners may have access to better service and support.	questionnaire	Quantitative	+	[58]
						Trust	$C_{4\_8}$	Trust based on respect, integrity and reciprocity is a key element in achieving cooperation.	questionnaire	Qualitative	+	[70,72]
						Reputation	$C_{4\_9}$	Reputation refers to public opinion about LSPs relates with service ability, social responsibility, innovation issues, etc. Good reputation is extremely crucial in the initial screening of LSPs	questionnaire	Qualitative	+	[7,8,12,46,53,57,67,68,69,70,72]
						Market share	$C_{4\_10}$	Sound financial performance ensures the continuity and sustainable development of operations. Indicators $C_{4\_10}$ to $C_{4\_19}$ cover profitability, operating ability, growth ability and debt servicing ability, obtained from the CSMAR database. ROI is used to measure the profitability of specific investments, such as fixed investment, R&D investment. Size of fixed assets, such as vehicles, warehouses, packing and labeling lines, and cold chain equipment, reflects the expertise and flexibility of the LSP, which is a plus point. LSPs with abundant logistics resources tend to establish a more complete logistics system. R&D investment refers to effort made for technological advancement, such as updating automation equipment, developing information platform, optimizing logistics network, and training employees.	LSP revenue/logistics market revenue	Quantitative	+	[2,56,72]
						Revenue	$C_{4\_11}$		annual average amount of contracts from primary operations	Quantitative	+	[54,56]
						Revenue growth rate	$C_{4\_12}$		$\frac{\mathrm{year-over-year}\;\mathrm{revenue}\;\mathrm{growth}}{\mathrm{revenue}\;\mathrm{from}\;\mathrm{the}\;\mathrm{previous}\;\mathrm{year}}$	Quantitative	+	[2,53]
						Return on equity (ROE)	$C_{4\_13}$		net profit/net asset	Quantitative	+	[2,53,56]
						Return on investment (ROI)	$C_{4\_14}$		financial statement	Quantitative	+	[12]
						Investment in fixed assets	$C_{4\_15}$		financial statement	Quantitative	+	[7,8,72,76]
						Investment growth rate in fixed assets	$C_{4\_16}$		$\frac{\mathrm{year-over-year}\;\mathrm{investment}\;\mathrm{growth}}{\mathrm{investment}\;\mathrm{from}\;\mathrm{the}\;\mathrm{previous}\;\mathrm{year}}$	Quantitative	+	[71]
						R&D investment ratio	$C_{4\_17}$		R&D investment/revenue	Quantitative	+	[2,12,46,53,56]
						Accounts receivable turnover ratio	$C_{4\_18}$		net revenue/average accounts receivable	Quantitative	+	[2]
						Asset liability ratio	$C_{4\_19}$		total liability/total asset	Quantitative	–	[2,12,16]

**Table A2:** The evaluation data of 4 alternative LSPs on 10 quantitative criteria.

	$C_{1}$	$C_{3}$	$C_{5}$	$C_{6}$	$C_{7}$	$C_{8}$	$C_{12}$	$C_{13}$
$A_{1}$	0.93	0.98	5	[2, 5]	50,000	1	1.15	50
$A_{2}$	0.96	1.00	2	[5, 7]	227,000	1	1.10	45
$A_{3}$	1.00	1.00	3	[2, 3]	170,000	1	1.05	48
$A_{4}$	0.60	1.00	1	[1, 9]	90,000	0	0.90	40
	$C_{2}$							
$A_{1}$	36, 38, 37, 33, 35, 37, 43, 42, 33, 42, 37, 36, 37, 36, 36, 39, 39, 39, 37, 34, 37, 39, 37, 38, 37, 35, 37, 34, 38, 34, 34, 34, 30, 39, 37, 34, 39, 33, 36, 36, 37, 37, 34, 36, 36, 37, 38, 38, 34, 36, 34, 34, 36, 39, 34, 37, 36, 38, 34, 36, 37, 38, 39, 36, 33, 35, 34, 41, 35, 37, 36, 38, 34, 33, 33, 37, 36, 36, 39, 37, 36, 39, 34, 37, 38, 36, 36, 34, 34, 36, 37, 41, 35, 36, 36, 32, 35, 32, 38, 34, 36, 35, 37, 35, 37, 37, 39, 36, 32, 34, 39, 34, 38, 36, 39, 32, 36, 34, 42, 38, 39, 34, 35, 35, 38, 35, 37, 32, 35, 34, 33, 37, 37, 36, 33, 38, 37, 35, 36, 35, 32, 35, 34, 34, 34, 35, 32, 38, 37, 36, 36, 34, 38, 36, 35, 39, 36, 35, 35, 34, 34, 41, 39, 37, 33, 34, 36, 38, 33, 31, 33, 37, 37, 37, 36, 36, 35, 38, 33, 37, 34, 35, 37, 38, 34, 39, 37, 36, 36, 36, 35, 36, 36, 38, 39, 37, 36, 37, 36, 34, 38, 37, 36, 37, 37, 34, 36, 36, 35, 39, 34, 35, 35, 34, 36, 35, 39, 36, 34, 39, 38, 36, 33, 35, 36, 37, 35, 37, 37, 33, 34, 35, 35, 35, 36, 30, 35, 38, 34, 38, 37, 36, 36, 33, 36, 39, 36, 36, 35, 36, 36, 37, 35, 36, 40, 31, 40, 37, 38, 33, 35, 35, 37}							
$A_{2}$	{51, 51, 50, 51, 52, 51, 52, 52, 50, 51, 50, 50, 52, 51, 51, 52, 52, 51, 52, 52, 51, 50, 50, 52, 49, 51, 49, 52, 52, 51, 51, 49, 52, 49, 49, 51, 50, 51, 52, 52, 50, 51, 51, 52, 52, 52, 51, 51, 52, 49, 50, 50, 51, 52, 52, 50, 51, 51, 51, 51, 51, 50, 51, 49, 52, 50, 50, 51, 50, 51, 50, 53, 52, 49, 51, 50, 51, 51, 52, 51, 53, 51, 51, 52, 51, 52, 51, 50, 49, 53, 50, 51, 52, 51, 50, 51, 51, 52, 50, 52, 51, 51, 50, 51, 51, 50, 51, 50, 51, 51, 50, 51, 51, 49, 52, 53, 52, 51, 51, 50, 53, 52, 52, 50, 51, 50, 51, 50, 50, 50, 51, 49, 52, 51, 52, 51, 51, 50, 50, 51, 51, 52, 51, 52, 51, 52, 51, 50, 50, 51, 52, 51, 50, 51, 50, 51, 51, 51, 51, 52, 51, 53, 51, 53, 50, 52, 51, 52, 52, 49, 50, 50, 51, 52, 51, 52, 52, 50, 51, 50, 53, 51, 53, 51, 52, 51, 49, 50, 52, 51, 50, 50, 51, 50, 52, 50, 53, 50, 51, 49, 50, 50, 51, 52, 51, 51, 52, 52, 51, 52, 51, 50, 52, 50, 50, 51, 51, 51, 52, 52, 51, 51, 51, 51, 51, 50, 51, 52, 51, 53, 50, 50, 49, 52, 52, 51, 52, 51, 51, 51, 51, 51, 54, 50, 49, 50, 50, 51, 51, 51, 52, 51, 52, 53, 52, 50, 49, 52, 49, 51, 51, 53, 51, 50, 51, 51, 51, 50, 50, 51, 50, 50, 52, 51, 51, 52, 51, 52, 51, 52, 52, 51, 50}							
$A_{3}$	{40, 39, 40, 40, 40, 40, 40, 41, 40, 40, 40, 40, 40, 40, 40, 41, 40, 40, 40, 40, 40, 41, 39, 41, 41, 40, 41, 40, 39, 39, 40, 40, 40, 40, 40, 40, 39, 40, 40, 40, 40, 39, 40, 40, 40, 40, 40, 40, 40, 40, 40, 40, 41, 40, 41, 40, 40, 41, 40, 40, 40, 40, 41, 41, 40, 40, 40, 39, 40, 40, 40, 40, 41, 40, 40, 40, 40, 39, 40, 41, 40, 40, 39, 40, 40, 39, 40, 40, 40, 40, 39, 39, 39, 40, 39, 39, 40, 39, 40, 40, 40, 40, 40, 39, 40, 40, 41, 40, 39, 40, 40, 40, 39, 41, 39, 40, 39, 40, 40, 40, 40, 40, 40, 40, 40, 41}							
$A_{4}$	{37, 50, 42, 43, 37, 31, 35, 33, 34, 40, 34, 33, 35, 36, 39, 32, 39, 37, 44, 36, 36, 31, 30, 39, 36, 40, 33, 47, 38, 42, 36, 36, 40, 40, 31, 29, 32, 30, 37, 35, 35, 30, 41, 39, 37, 42, 33, 39, 39, 39, 33, 39, 34, 33, 31, 42, 33, 36, 31, 36, 21, 32, 30, 32, 36, 35, 47, 34, 36, 29, 35, 30, 37, 41, 39, 33, 29, 44, 47, 38, 32, 43, 34, 35, 41, 30, 27, 39, 39, 37, 39, 42, 42, 34, 41, 38, 34, 31, 40, 35, 37, 33, 29, 38, 37, 43, 41, 42, 33, 32, 31, 39, 51, 41, 33, 41, 31, 30, 41, 33, 39, 44, 39, 42, 29, 40, 48, 40, 29, 36, 35, 39, 39, 39, 35, 34, 34, 41, 28, 36, 38, 34, 39, 37, 32, 38, 36, 35, 33, 37, 28, 35, 34, 32, 40, 33, 40, 42, 36, 38, 40, 37, 40, 31, 43, 32, 28, 44, 37, 39, 35, 32, 22, 40, 36, 36, 39, 30, 33, 43, 40, 31, 26, 34, 38, 42, 37, 31, 39, 31, 32, 34, 31, 36, 33, 36, 35, 35, 34, 43, 40, 37, 30, 29, 27, 50, 42, 38, 32, 31, 40, 45, 29, 35, 37, 27, 41, 35, 47, 35, 39, 31, 34, 31, 39, 44, 34, 39, 34, 38, 43, 38, 32, 33, 36, 36, 33, 38, 43, 36, 36, 26, 33, 30, 35, 40, 45, 46, 36, 35, 41, 39, 41, 41, 30, 40, 37, 33, 34, 43, 33, 31, 38, 47, 37, 39, 32, 45, 38, 40, 32, 41, 39, 31, 37, 35, 38, 36, 36, 32, 35, 41, 46, 31, 49, 45, 38, 35}							
	$C_{4}$							
$A_{1}$	{0.94, 0, 0, 0, 7.52, 2.29, 0.64, 0, 0, 7.15, 6.42, 0, 0, 8.16, 3.17, 8.15, 0, 0, 0, 0, 9.51, 0, 0.60, 0, 0, 0, 0, 2.24, 6.52, 6.05, 0, 0, 0, 0, 1.84, 7.26, 0, 8.42, 7.34, 0, 1.77, 9.57, 0, 9.25, 0, 0, 0, 6.40, 0, 0, 7.23, 3.47, 6.61, 0, 0, 0.22, 9.11, 8.01, 0, 8.13, 0, 0, 5.75, 5.30, 2.75, 0, 4.52, 0, 8.04, 9.86, 0.30, 0, 0, 0, 9.89, 0.67, 9.39, 0, 0, 0, 5.34, 0, 0, 6.26, 1.38, 2.18, 0, 0.42, 1.07, 0, 0, 0, 4.11, 0, 9.46, 6.77, 0, 0, 3.37, 6.62, 0, 0, 0, 0, 4.12, 6.03, 7.51, 0, 5.52, 0, 0, 0, 7.20, 0, 0, 0, 0, 0, 0, 0, 0, 0, 3.09, 7.26, 7.83, 0, 0.10, 0, 0, 0, 0, 0, 0, 0, 0, 0, 0, 4.77, 6.24, 2.36, 0, 8.30, 0, 0, 0, 0, 0, 0, 1.93, 0, 0, 0.44, 0, 7.72, 3.12, 1.79, 3.39, 0, 0, 9.06, 0, 0, 0, 0.55, 0, 0, 0, 0, 4.86, 8.94, 1.38, 3.90, 0, 0, 0, 0, 0, 9.36, 0, 7.31, 6.46, 0, 0, 0, 8.35, 3.22, 0, 9.79, 5.49, 3.30, 6.19, 0, 0, 0, 0, 0, 0, 3.28, 0, 7.39, 0, 0.32, 0, 0, 0, 0, 7.11, 0, 0, 0, 0, 0, 0, 2.41, 7.15, 0, 2.82, 0, 1.38, 0, 0, 0, 0, 0, 5.04, 4.90, 8.77, 3.53, 0, 0, 0, 0, 0, 6.67, 0, 6.75, 0, 0, 8.11, 0, 0, 0, 0, 0, 4.03, 0, 0, 2.58, 0, 0, 0, 1.22, 0, 0, 0, 0, 0, 3.42, 0, 0, 0, 0, 0}							
$A_{2}$	{2.51, 0, 0, 0, 0, 4.95, 2.44, 2.21, 0, 0, 0, 0, 0, 0, 0, 0, 0, 1.39, 0, 0, 0, 2.21, 0, 3.76, 0, 0, 0.68, 4.36, 0, 0.26, 9.55, 0, 0, 0, 0.07, 6.80, 0, 6.45, 5.52, 0, 0, 0, 0, 0, 0, 0, 0, 6.09, 9.10, 0, 0, 0, 0, 0, 0, 0.33, 0, 7.16, 0, 0, 0, 0, 0, 0, 0, 5.53, 2.75, 2.42, 0, 0, 0, 0, 8.19, 0, 0, 0, 1.89, 0, 3.16, 0, 0, 5.43, 0, 0, 0, 0, 0, 5.76, 7.48, 6.46, 0, 0, 0, 0, 0, 0, 6.72, 0, 0, 0, 0, 5.32, 0, 0, 9.06, 0, 0.25, 6.71, 0, 0, 0.57, 4.50, 0, 6.87, 0, 6.50, 0, 0, 0, 1.16, 0, 9.80, 2.85, 0, 9.62, 0, 1.93, 3.42, 9.33, 0, 0, 0, 3.97, 3.75, 1.31, 0, 0, 6.15, 0, 5.73, 0, 0, 4.48, 0, 0, 0, 0, 0, 0, 0, 0, 0, 0, 0, 0, 0, 6.20, 0, 1.73, 0.90, 2.55, 8.59, 0, 0, 0, 0, 5.76, 8.11, 0, 0, 0.90, 0, 0, 0, 0, 5.57, 5.29, 8.30, 0, 0, 0, 4.52, 0, 1.10, 1.10, 2.70, 0, 0, 0, 0, 0, 0, 0, 6.39, 2.55, 0, 0, 5.85, 0, 0.61, 5.85, 0, 0, 0, 0, 3.93, 8.27, 0, 2.08, 0, 0, 6.71, 5.71, 0, 1.48, 4.76, 0, 0, 0, 0, 0, 4.51, 0, 0, 0, 5.32, 0, 0, 3.29, 0, 0, 0, 0, 0, 0, 2.64, 7.59, 9.95, 0, 7.81, 0, 0, 8.02, 0, 7.29, 4.98, 8.09, 0, 0.73, 0, 0, 0, 0, 7.49, 0, 0, 7.64, 0, 1.84, 0, 5.18, 9.94, 0, 0, 0, 0, 9.35, 0, 2.32, 3.96, 0, 0, 0, 9.95, 9.62, 5.35, 0, 0, 0.51, 0, 5.80, 0, 0}							
$A_{3}$	{0, 0, 0, 1.58, 0.89, 0, 0, 0, 2.49, 2.56, 0, 0, 0, 0, 0, 0, 0, 1.24, 0, 0, 0, 0, 0, 0, 0, 0, 0, 0, 0, 0, 0, 0, 0, 0, 0, 0, 0, 3.92, 5.56, 3.29, 0, 2.34, 0, 0, 0, 0, 0, 0, 0, 0, 6.54, 0, 0, 0, 0, 0, 0, 0, 0, 4.85, 0, 0, 0, 0, 0, 0, 0, 0, 0, 0, 0, 0, 0.35, 5.49, 5.87, 0, 3.04, 0, 0, 0, 0, 2.61, 0, 0, 0, 0, 0, 5.66, 0, 6.97, 0, 0, 0, 0, 0, 1.72, 0, 4.46, 0, 0, 7.21, 3.36, 0, 0, 2.05, 0, 3.73, 0, 3.45, 0, 0, 0, 0, 0, 0, 1.77, 0, 4.18, 0, 0, 0.56, 0, 5.44, 0, 0, 1.60}							
$A_{4}$	{4.20, 0, 4.72, 0, 0, 0, 0, 0, 0.25, 0, 0, 0, 0, 0, 0, 0, 1.28, 4.43, 0, 4.08, 0, 0, 0, 4.50, 0, 0, 0, 0, 3.53, 0, 0, 3.79, 0, 0, 0, 0, 0, 3.93, 0, 1.39, 0, 0, 0, 0, 0, 1.39, 0, 0, 0, 0, 0, 0, 0, 0, 2.17, 0, 0.97, 0, 1.35, 0, 0, 3.57, 0, 0, 0, 3.04, 4.74, 0, 1.34, 0, 0, 0, 0, 0, 1.90, 0, 0, 0, 2.36, 0, 4.88, 0, 0, 0, 0, 0, 0, 2.62, 0, 3.70, 0, 0, 0, 4.80, 0, 0, 0, 0, 0.07, 0, 2.36, 0, 0, 0, 0, 0, 2.19, 0, 0, 3.04, 0, 0, 0, 0, 0, 0, 0, 0, 4.37, 0, 0, 4.50, 1.09, 0, 0, 4.18, 2.35, 2.07, 0, 0, 0, 0, 3.01, 0, 0, 0.29, 0, 0, 0, 0, 0, 0, 0, 0.35, 0, 1.90, 0, 0, 1.20, 0, 2.40, 0, 0, 0, 0, 0, 0, 0.33, 0, 0.02, 0, 2.54, 0, 0, 0, 0, 0.34, 0.42, 0, 0, 0, 0, 4.49, 0, 0, 0, 4.38, 1.74, 0, 0, 0.38, 0, 0, 0, 0, 0, 0, 0, 3.31, 0, 0, 0, 0.34, 0, 0, 0, 0, 2.66, 0, 0, 0, 0, 0, 0, 0.59, 0, 3.42, 0, 4.85, 0, 0, 0, 2.01, 0, 0, 3.26, 0, 0, 0, 0, 0, 0, 0.90, 0, 4.53, 0, 0, 0, 0.18, 0, 0, 2.56, 0, 0, 4.94, 4.61, 0, 0, 0, 0, 0, 1.87, 4.61, 0, 0, 2.48, 1.54, 0, 0, 0, 4.96, 0, 0, 0, 0.97, 0, 3.63, 0, 0, 0, 0, 0, 0, 0, 0.26, 0, 0, 1.10, 2.03, 0, 0, 0, 0, 0, 2.09, 2.44, 0, 0, 0, 0.60, 0, 0, 0, 2.47, 0, 0, 0, 4.07}							

**Table A3:** The evaluation data of 4 alternative LSPs on 6 qualitative criteria.

	$C_{9}$						
	$DM_{1}$	$DM_{2}$	$DM_{3}$	$DM_{4}$	$DM_{5}$	$DM_{6}$	$DM_{7}$
$A_{1}$	8	[7, 8]	between l(9,5) and l(9,8)	l(3,2)	greater than l(7,4)	l(5,3)	l(5,4)
$A_{2}$	7	l(5,3)	at least l(9,7)	l(3,3)	[7.5, 8]	10	greater than l(7,6)
$A_{3}$	6	at most l(5,2)	l(9,4)	l(3,2)	l(7,3)	8	lower than l(7,5)
$A_{4}$	7	l(5,4)	l(9,6)	l(3,1)	[3, 5]	[8.5, 9.5]	l(7,6)
	$C_{10}$						
	$DM_{1}$		$DM_{2}$		$DM_{3}$		$DM_{4}$
$A_{1}$	9		8.5		l(7,6)		between l(5,3) and l(5,5)
A2	between l(5,4) and l(5,5)		l(7,6)		10		l(5,4)
$A_{3}$	l(5,4)		7		9.5		l(5,5)
$A_{4}$	5		lower than l(7,3)		[2, 3]		at most l(5,3)
	$C_{11}$						
	$DM_{1}$		$DM_{2}$	$DM_{3}$	$DM_{4}$	$DM_{5}$	
$A_{1}$	l(7,5)		l(5,4)	at least l(7,5)	[7, 8]]	l(9,6)	
$A_{2}$	8		l(5,5)	l(7,6)	9.5	between l(7,4) and l(7,5)	
$A_{3}$	8.5		l(5,4)	l(7,6)	8	at least l(7,5)	
$A_{4}$	at least l(7,6)		greater than l(5,3)	l(7,5)	l(3,2)]	l(5,2)	
	$C_{14}$				$C_{15}$		
	$DM_{1}$	$DM_{2}$	$DM_{3}$	$DM_{4}$	$DM_{1}$	$DM_{2}$	$DM_{3}$
$A_{1}$	l(7, 6)	[6, 8.5]	l(5,4)	between l(9,4) and l(9,5)	at least l(7,5)	8.5	l(3,2)
$A_{2}$	l(5,3)	8	greater than l(7,6)	l(9,6)	between l(7,3) and l(7,6)	l(5,5)	l(3,3)
$A_{3}$	at least l(7,5)	9	l(5,5)	greater than l(9,7)	lower than l(7,5)	l(5,2)	[8, 9.5]
$A_{4}$	[3.5, 5.5]	5	6	at most l(5,4)	l(7,6)	at most l(5,3)	7
	$C_{16}$						
	$DM_{1}$		$DM_{2}$		$DM_{3}$		$DM_{4}$
$A_{1}$	at least l(7,4)		8		l(7,6)		[5.5, 7.5]
A2	l(7,4)		3		lower than l(7,4)		[4.5, 5]
$A_{3}$	l(7,7)		between l(5,4) and l(5,5)		l(7,5)		l(5,3)
$A_{4}$	l(7,5)		6		greater than l(5,3)		l(5,1)
